# Will previous antimicrobial therapy reduce the positivity rate of metagenomic next-generation sequencing in periprosthetic joint infections? A clinical study

**DOI:** 10.3389/fcimb.2023.1295962

**Published:** 2024-01-11

**Authors:** Linjie Hao, Weiguo Bian, Zhong Qing, Tao Ma, Hui Li, Peng Xu, Pengfei Wen

**Affiliations:** ^1^ Department of Joint Surgery, Honghui Hospital, Xi’an Jiaotong University, Xi’an, China; ^2^ Department of Orthopedics, The First Affiliated Hospital of Xi’an Jiaotong University, Xi’an, China

**Keywords:** metagenomic next-generation sequencing, antibiotics, periprosthetic joint infection, pathogen detection, positivity rate

## Abstract

**Background:**

Metagenomic next-generation sequencing (mNGS) is a culture-independent massively parallel DNA sequencing technology and it has been widely used for rapid etiological diagnosis with significantly high positivity rate. Currently, clinical studies on evaluating the influence of previous antimicrobial therapy on positivity rate of mNGS in PJIs are rarely reported. The present study aimed to investigate whether the positivity rate of mNGS is susceptible to previous antimicrobial therapy.

**Methods:**

We performed a prospective trial among patients who undergone hip or knee surgery due to periprosthetic joint infection (PJI) to compare the positivity rate of culture and mNGS between cases with and without previous antimicrobial therapy, and the positivity rates between cases with different antimicrobial-free intervals were also analysed.

**Results:**

Among 131 included PJIs, 91 (69.5%) had positive cultures and 115 (87.8%) had positive mNGS results. There was no significant difference in the positivity rate of deep-tissue culture and synovial fluid mNGS between cases with and without previous antimicrobial therapy. The positivity rate of synovial fluid culture was higher in cases with previous antimicrobial therapy. The positivity rates of mNGS in synovial fluid decreased as the antimicrobial-free interval ranged from 4 to 14 days to 0 to 3 days.

**Conclusion:**

mNGS is more advantageous than culture with a higher pathogen detection rate. However, our data suggested that antimicrobial agents may need to be discontinued more than 3 days before sampling to further increase the positivity rate of mNGS for PJIs.

## Introduction

Periprosthetic joint infection (PJI) is one of the most serious complications following total joint arthroplasty (TJA) (Koh et al., 2017), with a significant impact on patients’ quality of life (Helwig et al., 2014). Synovial fluid culture is one of the most commonly used methods to detect the pathogen(s) for PJI, and intraoperative culture of periprosthetic tissue is considered the gold standard for the microbiological diagnosis of PJI (Osmon et al., 2013). To date, the negative detection rate of pathogens for PJI by culture is consistently no less than 20%~40%, and the undetected polymicrobial infection has not yet been taken into account (Parvizi et al., 2014). Reasons for false-negative results are mainly the presence of biofilms, low bacterial load in chronic low-grade infections and previous antibiotic administration (Malekzadeh et al., 2010). Therefore, it has been recommended to discontinue antimicrobial therapy at least 2 weeks prior to sampling (Trampuz et al., 2007).

Metagenomic next-generation sequencing (mNGS) is a culture-independent, massively parallel DNA sequencing technology that has been widely used for rapid aetiological diagnosis with significantly high positivity rates (Wilson et al., 2014; Xiao et al., 2019; Diao et al., 2021; Zhao et al., 2021). Theoretically, mNGS is insensitive to prior antimicrobial therapy because the nucleic acids of dead microorganisms are still detectable. However, the half-life of cell-free DNA from lytic microorganisms is short, which can lead to low detectable pathogen sequences and false negative results. Grumaz et al. (2016) showed that the number of pathogen sequences decreased significantly after a few days of antimicrobial therapy in patients with toxaemia, indicating that previous antimicrobial therapy may have an adverse effect on mNGS. A study by Miao Q et al, which included 510 blood samples from patients suspended with infectious diseases, found that mNGS could provide higher sensitivity for pathogen identification and was less affected by prior antibiotic exposure (Miao et al., 2018). Currently, clinical studies evaluating the influence of prior antimicrobial therapy on the positivity rate of mNGS in PJI are rarely reported. Meanwhile, it is not clear whether suspension of antimicrobial therapy within 14 days prior to specimen collection (in terms of culture) is appropriate for mNGS. In this study, we report the results of a prospective analysis comparing the positivity rates of mNGS and culture in PJIs with different antimicrobial-free intervals before sampling and explore the optimal antimicrobial-free interval for mNGS.

## Materials and methods

### Study population

**Figure 1:**
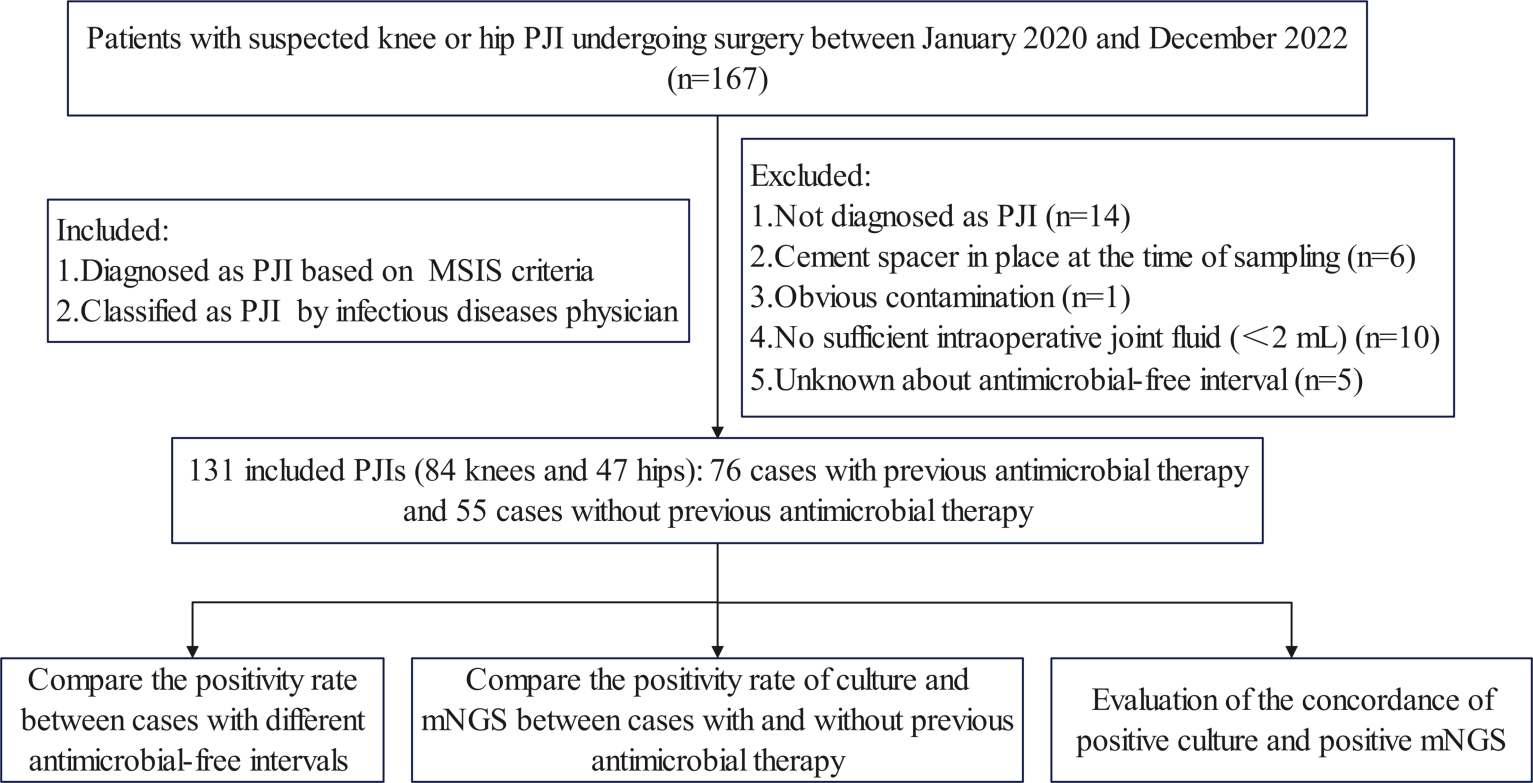
Flowchart detailing the procedure of this study.

The present study was a prospective, single-centre clinical trial conducted in accordance with the World Medical Association Declaration of Helsinki and approved by the Ethics Committee of Honghui Hospital Affiliated to Xi’an Jiao Tong University. Written informed consent was obtained from all subjects. Patients with suspected knee or hip PJI who underwent surgery at our institution between January 2020 and December 2022 were included. Patients were diagnosed with PJI based on the diagnostic criteria of the Musculoskeletal Infection Society (MSIS) (Parvizi et al., 2011). Six cases that did not meet the MSIS PJI diagnostic criteria were classified as PJI after blinded review of all preoperative and intraoperative information by an infectious disease physician. Patients who were not diagnosed with PJI after revision surgery were excluded. Meanwhile, PJIs were excluded if a cement spacer was in place at the time of sampling, if there was obvious contamination, if the amount of joint fluid (<2 mL) was insufficient for both culture and mNGS testing, or if the antimicrobial-free interval was unknown (**Figure 1**).

Diagnostic data including serum C-reactive protein (CRP), serum erythrocyte sedimentation rate (ESR), synovial fluid white blood cell (WBC) count and polymorphonuclear neutrophil (PMN) percentage, and microbiological results were collected. Other data including patient age and sex, prosthesis site (hip or knee), type of infection (acute or chronic), and previous antimicrobial therapy including antimicrobial-free interval were collected. The positivity rate of culture and mNGS was compared between cases with and without previous antimicrobial therapy, and the positivity rate between cases with different antimicrobial-free intervals was also analysed.

### Sampling

Synovial fluid and deep tissue were obtained at the time of surgery. Synovial fluid was collected in a sterile manner using an 18-gauge needle prior to arthrotomy, and samples were immediately divided into aerobic and anaerobic blood culture bottles (BACTEC 9240 system; BD Diagnostic Systems) for microbiology, a sterile container free of nucleases or other amplification inhibitors for mNGS, and an ethylenediaminetetraacetic acid (EDTA) vial for white blood cell count and differential. At least three different deep tissue samples were taken from periprosthetic tissue with the most obvious inflammatory changes and from medullary canals. Deep tissue samples were immediately divided into separate sterile vials for culture and histological analysis (more than 5 neutrophils per high-power field in 5 high-power fields observed was positive). All samples for microbiological testing were processed within 4 hours. All cases with at least 1 positive culture were considered positive.

### Culture

Deep tissue samples were homogenised and routine cultures, including aerobic and anaerobic bacterial cultures, fungal cultures and acid-fast bacilli cultures, were performed simultaneously. For synovial fluid culture, blood culture bottles were incubated on the BD-BACTEC-9240 instrument (BD Diagnostic Systems) for a total of 3 weeks if negative. Positive blood culture bottles were Gram stained and subcultured. Bacterial identification and drug susceptibility were performed using a Vitek II system (Biomerieux, USA). Quantitative culture methods were used from the samples and appropriate negative controls were established to avoid the adverse effect of contamination.

### mNGS detection

The workflow for mNGS in synovial fluid included sample preparation, nucleic acid extraction, DNA library construction, metagenomic sequencing and bioinformatic analysis. Full details are available in **Supplementary Appendix 1**. DNA extraction was performed using the TIANamp Micro DNA Kit (DP316, TIANGEN Biot ech, China). DNA library construction was performed using the MGIEasy FS DNA Library Prep Kit (MGI Tech, China). Sequencing was performed on the BGISEQ-500 platform (BGI-Tianjin, China). A negative control (sterile water) and a positive control of a known pathogen were run on the same batch of samples. If obvious contamination was found, the sample was retested. The pathogens detected by mNGS were considered positive after screening according to the predetermined threshold and after the contamination was eliminated. The pre-defined thresholds are as follows. (i) Bacteria (except mycobacteria): Organisms with a coverage rate 10 times or more than the other organisms were considered to be the pathogenic species. For an organism that did not match the negative control pathogen, it was considered to be the pathogenic species if the number of reads stringently mapped to the pathogen at the genus level was ≥10. For an organism that matched the negative control pathogen, it was considered positive if the coverage rate was ≥2% and the number of reads stringently mapped to pathogen at the genus level was ≥10 in two consecutive tests. (ii) Fungi and viruses: Due to low nucleic acid yield, if the number of reads mapped to pathogen at genus or species level ≥10 and in the top 10 for bacteria, they were considered positive. (iii) Mycobacterium: Due to the extremely low nucleic acid yield, if the number of reads stringently mapped to pathogen at genus level ≥1 and the number was in the top 10, it was considered to be the pathogenic species. (iv) Parasites were generally not considered to be pathogens. (v)*Burkholderia, Ralstonia, Delftia, Sphingobium, Alternaria, Sodaria, Aspergillus, Albugo* and other genera were the most common background bacteria and were also detected in other sample types in our laboratory.

### Data and statistical analysis

Patients with PJI were divided into acute and chronic infections according to the duration of infection, as described by Talsma et al. (2021). Acute PJIs were defined as either early acute/postoperative infections (i.e. within 3 months of the index arthroplasty) or late acute/haematogenous infections, both with a symptom duration of less than 3 weeks before re-operation. Chronic PJI was defined as late infections (i.e., more than 3 months after the index arthroplasty) with a symptom duration of more than 3 weeks. Previous antimicrobial therapy was defined as receipt of antimicrobials within 4 weeks prior to reoperation. The antimicrobial-free interval was divided into three subsets: more than 14 days (within 4 weeks), 4 to 14 days, and 0 to 3 days prior to sampling.

To assess the concordance of microbiological results between positive culture and positive mNGS, categorisation was determined as follows: (i) complete concordance: identical organism detected, (ii) partial concordance: additional organism detected by culture or additional organism detected by mNGS, and (iii) discordance: different organism detected.

Statistical analysis was performed using SPSS software (version 24, IBM Inc., Armonk, New York). Demographic data were described as mean and standard deviation (SD). Categorical variables were expressed as percentages. Student’s t-test was used to calculate differences between groups for continuous variables. Positive rates were calculated for microbiological and non-microbiological diagnostic tests. Chi-squared test or Fisher’s exact test was used to measure positivity rates between groups, as appropriate. McNemar’s test was used to compare mNGS and culture of samples within groups. The rank sum test was used to compare the number of mNGS pathogen sequencing reads between groups. Statistical significance was defined as p<0.05 (for two-tailed test).

## Results

### General characteristics

Of the 167 screened cases with suspected hip or knee PJI, 36 cases were excluded due to aseptic failure (n=14), no culture or mNGS testing of synovial fluid (n=10), cement spacers in place at the time of sampling (n=6), unknown antimicrobial-free interval (n=5), and contamination in the operating room (n=1). The remaining 131 cases (84 knees and 47 hips) were included, including 76 cases with previous antimicrobial therapy (**Supplementary Appendix 2**) and 55 cases without previous antimicrobial therapy (**Supplementary Appendix 3**). The mean time from primary joint replacement to this surgical procedure was 32.9 (range 0.6-94) months. Serum CRP and ESR results were available for all 131 cases. Synovial fluid WBC count and PMN percentage results were available for 62 patients with prior antimicrobial therapy and 47 patients without prior antimicrobial therapy. Positive histology results were available for 67 patients with prior antimicrobial therapy and 48 patients without prior antimicrobial therapy. Patient demographics and baseline data are shown in **Table 1**. The groups were similar in terms of age, sex ratio and distribution of joint type.

**Table 1:** Patient demographic and baseline data of 131 PJIs.

Characteristic	With antibiotics (N=76)	Without antibiotics (N=55)	p
Age^*^ (years)	65.5 ± 9.4	64.6 ± 13.5	0.631
Sex **^#^** (male/female)	47/29	30/25	0.402
Joint			0.115
Hip^†^	23 (30.3%)	24 (43.6%)	
Knee^†^	53 (69.7%)	31 (56.4%)	
Side			0.108
Left	28	48	
Right	28	27	
WBC (10^3^ cells/µL)^*^	9.2 ± 5.2	8.1 ± 3.8	0.195
MSIS criteria			
Major criteria^†^			
2 accordant positive-culture	40 (52.6%)	27 (49.1%)	0.689
Presence of sinus tract	13 (17.1%)	5 (9.1%)	0.189
Minor criteria			
Serum CRP (mg/L)^*^	60.7 ± 80.6	46.8 ± 63.6	0.289
Serum ESR (mm/h)^*^	47.0 ± 29.7	40.5 ± 28.4	0.205
Synovial WBC count (cells/µL)^*^	41394.6 ± 58081.1	40332.6 ± 83625.4	0.938
Synovial PMN percentage(%)^*^	82.3 ± 18.3	77.1 ± 26.8	0.229
Purulence visible intraoperatively^†^	45 (59.2%)	33 (60.0%)	0.928
A single positive-culture^†^	9 (11.8%)	4 (7.3%)	0.388
Positive histology^†^	59 (88.1%)	41 (85.4%)	0.678
Classification according to duration of infection^†^			0.020
Acute infection	39 (51.3%)	17 (30.9%)	
Chronic infection	37 (48.7%)	38 (69.1%)	

*The values indicate mean and standard deviation. #The values indicate the number of cases. †The values are the number of cases, with the percentage in parentheses. PJI, Periprosthetic joint infection; MSIS+, MSIS positive; MSIS-, MSIS negative; CRP, C-reactive protein; ESR, Erythrocyte sedimentation rate; WBC, White blood cell; PMN, Polymorphonuclear neutrophil.

### Diagnostic performance of non- and microbiologic tests

The positivity rates of all non-microbiological and microbiological diagnostic tests are shown in **Table 2**. Non-microbiological multimodal analysis (i.e., elevated serum CRP or ESR or elevated synovial WBC count or elevated PMN percentage and/or positive histology) established the diagnosis in 127 of 131 (96.9%) cases, whereas microbiological diagnostic evaluation (≥2 positive identical cultures and/or positive sequencing results) confirmed PJI pathogens in 116 of 131 (88.5%) cases.

**Table 2:** Positivity rates of non- and microbiologic tests.

Diagnostic tests	All cases (N=131)
Non-microbiologic tests	127/131 (96.9)
Elevated Serum CRP (>10mg/L)	106/131 (80.9)
Elevated Serum ESR (>30mm/h)	82/131 (62.6)
Elevated Synovial WBC (>3000 cells/µL)	90/109 (82.6)
Elevated Synovial PMN (>70%)	89/109 (81.7)
Positive histology	100/115 (87.0)
Microbiologic tests	116/131 (88.5)
Positive SF culture	66/131 (50.4)
DT culture (≥2 positive cultures)	59/131 (45.0)
DT culture (≥1 positive culture)	81/131 (61.8)
All positive cultures from SF and DT	91/131 (69.5)
Positive mNGS	115/131 (87.8)

The results are shown as numbers of cases (percentages). CRP, C-reactive protein; ESR, Erythrocyte sedimentation rate; PMN, Polymorphonuclear neutrophil. SF, Synovial fluid; DT, Deep tissue. mNGS, Metagenomic next-generation sequencing.

Of the 131 cases, 66 (50.4%) had positive synovial fluid cultures, 59 (45.0%) had two or more positive deep tissue cultures, 81 (61.8%) had one or more positive deep tissue cultures, and 22 (16.8%) had a single positive deep tissue culture. In addition, of the 22 cases with single positive deep tissue cultures, 17 (77.3%) were found to have identical pathogens in synovial fluid culture and/or mNGS testing. In the remaining 5 cases, the pathogen detected in the single positive tissue culture did not correlate with the pathogen detected in the synovial fluid cultures or mNGS tests, suggesting the possibility of contamination. When combining positive synovial fluid cultures and positive deep tissue cultures, the overall positivity rate was 69.5% (91 of 131 cases), which was a significantly lower percentage compared to the positivity rate of mNGS (115 of 131 cases [87.8%], p < 0.001). When compared with all cultures from all sources (deep tissue and synovial fluid), mNGS was able to identify identical pathogens in 90.1% (82/91) of culture-positive PJIs, with additional potential pathogens detected in 15.4% (14/91). Potential pathogens were detected in 70.0% (28/40) of culture-negative PJIs.

### Influence of previous antimicrobial therapy on microbial results

In the group with prior antimicrobial therapy, the positivity rate of mNGS was 88.2% (67 of 76), which was significantly higher than that of all cultures in all courses (synovial fluid and deep tissue) (58 of 76 [76.3%], p=0.004). In the group without prior antimicrobial therapy, the positivity rate of mNGS was 87.3% (48 of 55), which was significantly higher than that of all cultures in all courses (synovial fluid and deep tissue) (33 of 55 [60.0%], p= 0.013).

**Figure 2:**
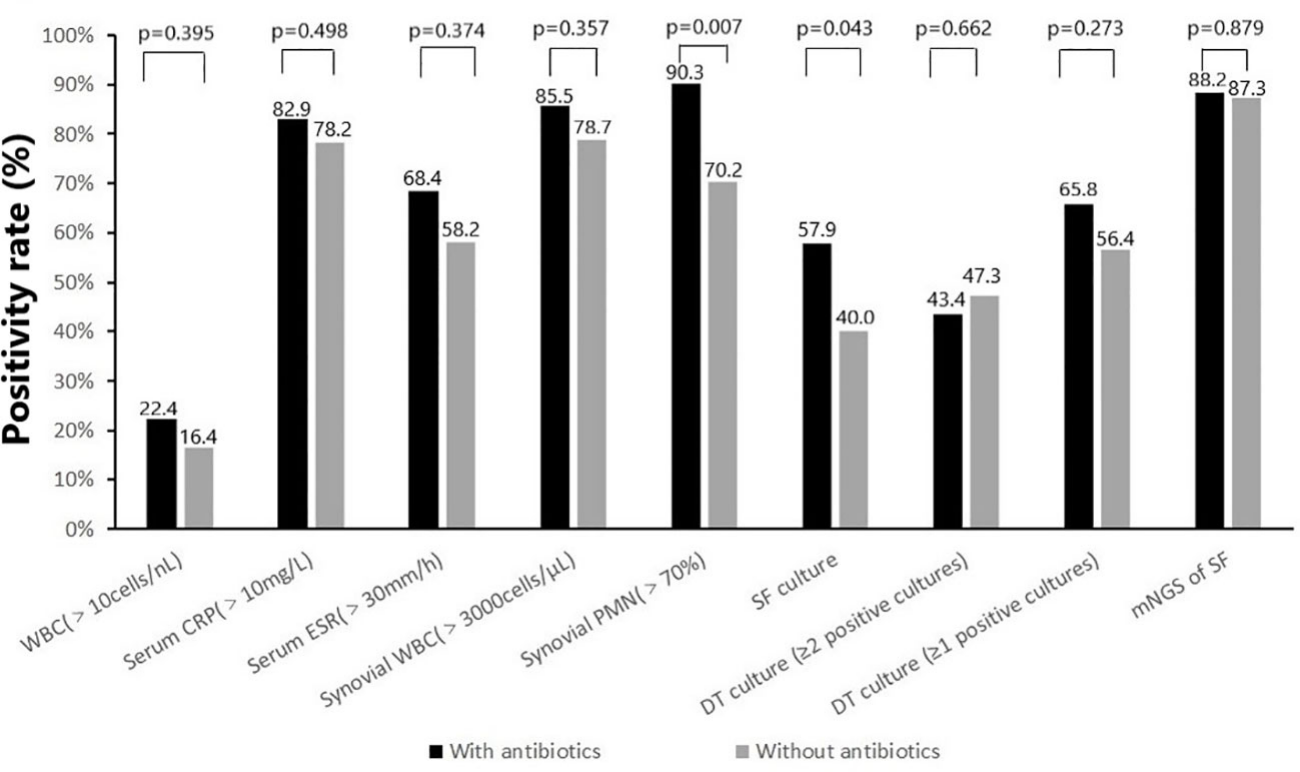
Positivity rate of (non-) microbiologic tests according to prior antimicrobial therapy: with antibiotics (N=76) and without antibiotics (N=55).

Almost all non-microbiological and microbiological tests showed a higher positivity rate (with or without statistical difference) in the group with prior antimicrobial therapy compared to the group without prior antimicrobial therapy (**Figure 2**). For non-microbiological tests, there were no significant differences in elevated WBC (17 of 76 [22.4%] vs 9 of 55 [16.4%]), elevated serum CRP (63 of 76 [82.9%] vs 43 of 55 [78. 2%]), elevated serum ESR (50 of 76 [68.4%] vs 32 of 55 [58.2%]) and elevated synovial WBC count (53 of 62 [85.5] vs 37 of 47 [78.7%]) between cases with and without previous antimicrobial therapy. While the positivity rate for elevated synovial PMN percentage (>70%) was significantly higher in cases with previous antimicrobial therapy compared to those without antibiotics (56 of 62 [90.3%] vs 33 of 47 [70.2%], p=0.007). For microbiological tests, there were no significant differences in the positivity rate of deep tissue culture (≥1 positive cultures [50/76, 65.8% vs 31/55, 56.4%] or ≥2 positive cultures [33/76, 43.4% vs 26/55, 47.3%]) and mNGS (67 of 76 [88.2%] vs 48 of 55 [87.3%]) between cases with and without previous antimicrobial therapy. However, the synovial fluid culture positivity rate was significantly higher in cases with previous antimicrobial therapy compared to cases without antibiotics (44 of 76 [57.9%] vs 22 of 55 [40.0%], p=0.043).

**Figure 3:**
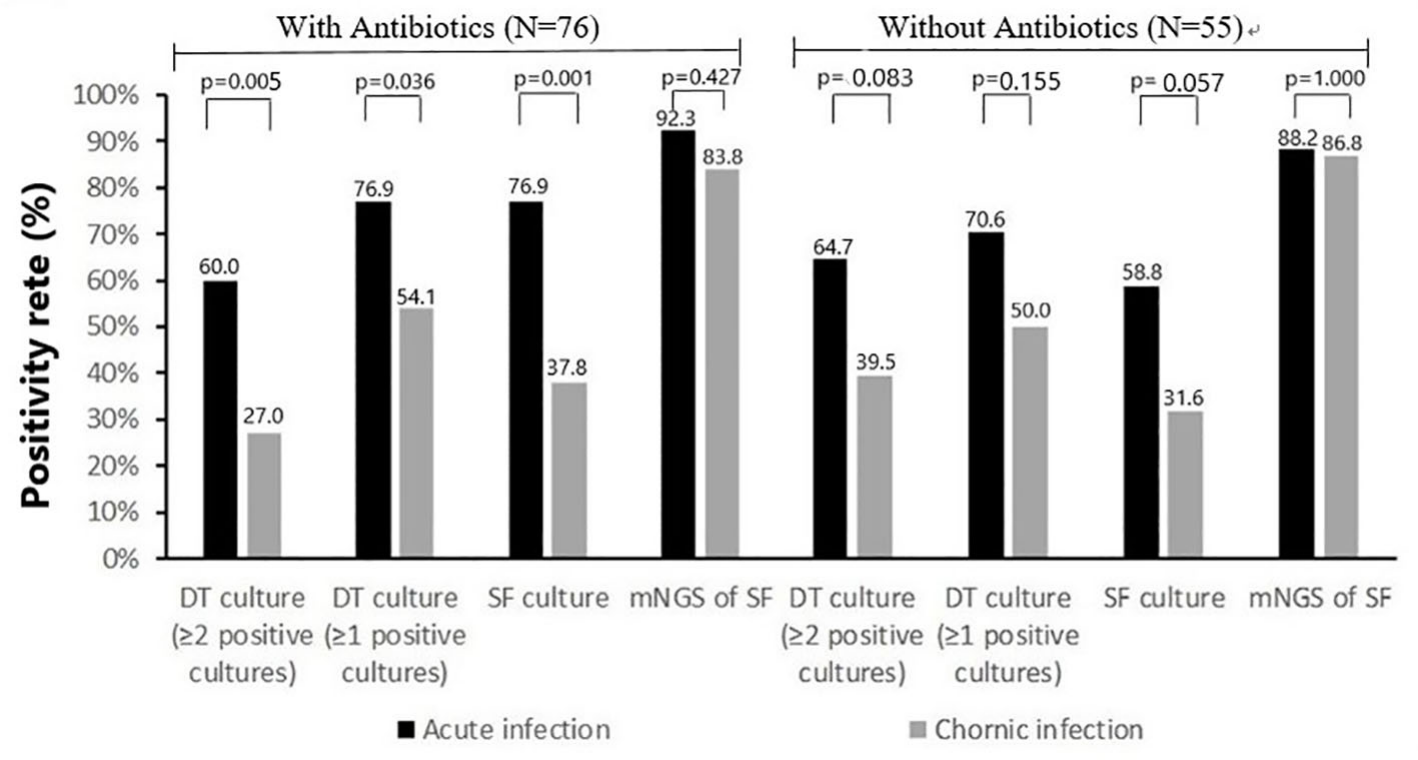
Positivity rate of microbiologic tests according to classification of infection: acute infection (N=56) and chronic infection (N=75).

The rate of previous antimicrobial therapy was significantly higher in acute PJIs (39 of 56 cases [69.6%]) than in chronic PJIs (37 of 75 cases [49.3%], p=0.020). Comparison of positivity rate of microbiologic tests between acute infections and chronic infections in separate group with or without previous antimicrobial therapy are shown in **Figure 3**. In the group without previous antimicrobial therapy, the positivity rate of deep-tissue culture (≥1 positive cultures or ≥2 positive cultures) and synovial fluid culture in chronic infections were lower than that of acute infection (19 of 38 [50.0%] vs 12 of 17 [70.6%], 15 of 38 [39.5%] vs 11 of 17 [64.7%], 12 of 38 [31.6%] vs 10 of 17 [58.8%], respectively), but the differences were not statistically significant (p>0.05). With the influence of previous antimicrobial therapy, the positivity rate of deep-tissue culture (≥1 positive cultures or ≥2 positive cultures) and synovial fluid culture in chronic infections were all significantly lower than that of acute infection (20 of 37 [54.1%] vs 30 of 39 [76.9%], 10 of 37 [27.0%] vs 23 of 39 [59.0%], 14 of 37 [37.8%] vs 30 of 39 [76.9%], respectively). While, positivity rate of mNGS in synovial fluid was similar in both groups (with previous antimicrobial therapy 36 of 39 [92.3%] vs 31 of 37 [83.8%], without previous antimicrobial therapy 15 of 17 [88.2%] vs 33 of 38 [86.8%]).

In relation to the sequencing read counts which was regarded as a semi-quantitative indicator of the detected pathogens abundance, the average number of reads for the top known pathogen identified in cases with previous antimicrobial therapy and without previous antimicrobial therapy was comparable (453.4 ± 1407.3 vs 428.3 ± 660.2, p=0.079), as was that for acute PJIs in both groups (643.8 ± 1797.5 with previous antimicrobial therapy vs 685.1 ± 1015.1 without previous antimicrobial therapy, p=0.289). While, the average number of reads for the top known pathogen identified in chronic PJIs was significantly higher in cases without previous antimicrobial therapy than cases with previous antimicrobial therapy (311.5 ± 380.1 vs 226.1 ± 669.4, p=0.033).

### Influence of different antimicrobial-free intervals on microbial results

**Figure 4:**
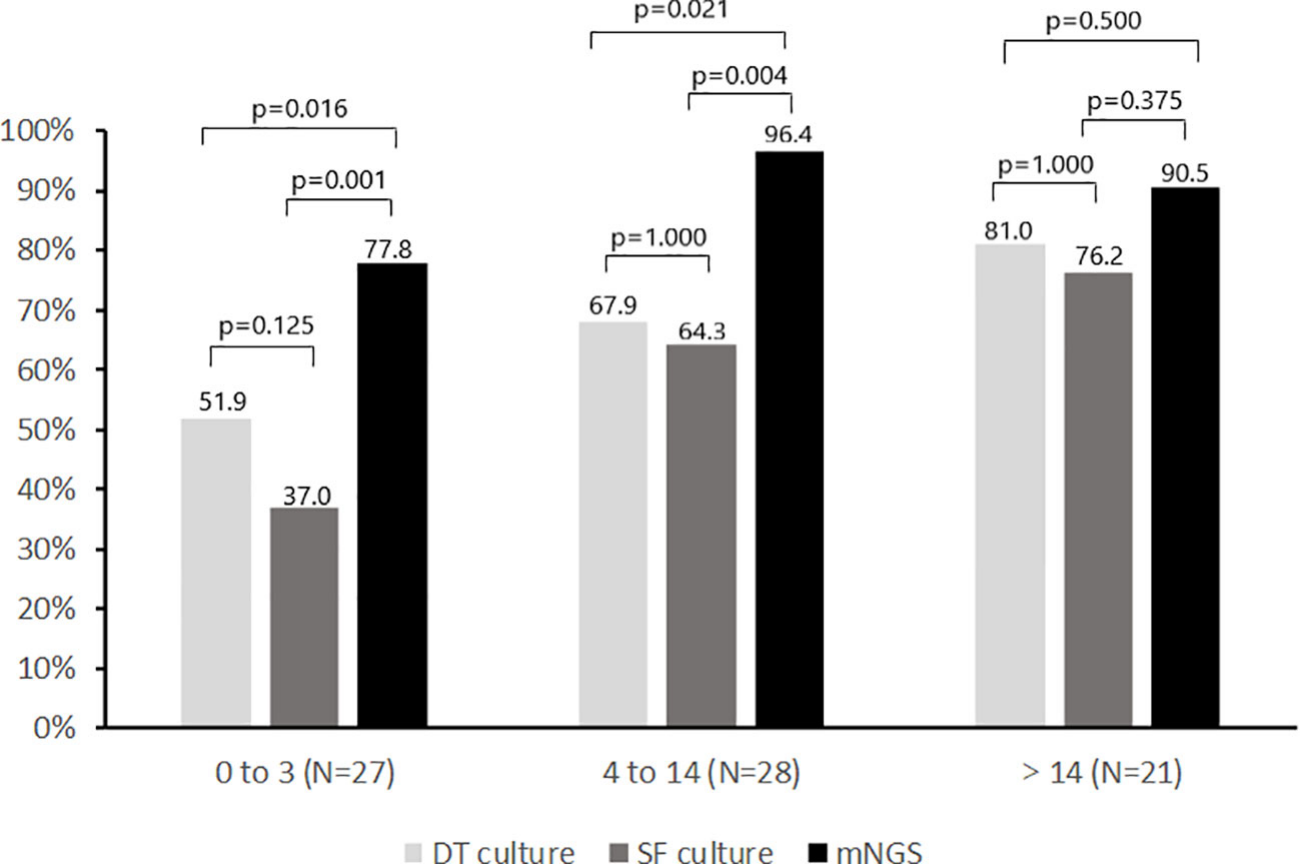
Positivity rate of microbiologic tests according to no. of days before surgery when antimicrobial therapy was discontinued. SF, Synovial fluid; DT, Deep tissue.

Comparison of the positivity rate of deep tissue culture (≥1 positive culture), synovial fluid culture and mNGS during different antimicrobial-free intervals is shown in **Figure 4**. The positivity rate of deep tissue and synovial fluid cultures decreased with shortening of the antimicrobial-free interval in patients receiving antimicrobial therapy prior to surgery. For deep tissue culture, the positivity rate decreased from 81.0% to 67.9% to 51.9% as the preoperative antimicrobial-free interval decreased from greater than 14 days, to 4 to 14 days, to 0 to 3 days, respectively (p for trend = 0.035). For synovial fluid culture, the positivity rate decreased from 76.2% to 64.3% to 37.0% over the same time intervals (p for trend = 0.006). For mNGS, the positivity rates were 90.5%, 96.4% and 77.8% for the same time intervals (p for trend = 0.142). Although the positivity rate of mNGS decreased as the antimicrobial-free interval before surgery decreased from 4-14 days to 0-3 days (p = 0.051), it was still higher than that of deep tissue and synovial fluid cultures (p<0.05). Furthermore, the positivity rate of mNGS was higher than that of all cultures from all sources (deep tissue and synovial fluid) when antimicrobial therapy was stopped within 14 days before surgery (48 of 55 [87.3%] vs 38 of 55 [69.1%], p=0.013).

### Concordance of microbial results

The pathogens based on their frequency of isolation by culture and detection by mNGS are shown in **Table 3**. When comparing all cultures from all sources (deep tissue and synovial fluid) in cases with previous antimicrobial therapy, 12 cases (15.8%) were positive for mNGS but negative for culture, three cases (3.9%) were negative for mNGS but positive for culture, six cases (7.9%) had identical negative results, and 55 cases (72.4%) had both positive culture and mNGS results. Of the 55 double-positive cases, there was complete concordance between culture and mNGS results in 44 cases (80.0%). In 11 cases, there was partial concordance (8 of 55 [14.5%]) or discordance (3 of 55 [5.5%]) of the microbial findings (**Table 4**).

**Table 3:** Correspondence between culture growth and the pathogen detected by mNGS.

Pathogen	Culture*	mNGS*
Coagulase-negative Staphylococcus^a^	45	50
*Staphylococcus aureus*	13	18
*Streptococcus* spp.^b^	12	16
*Propionibacterium* spp.^c^	8	10
*Enterococcus* spp.^d^	8	8
Fungus^e^	3	6
*Bacillus cereus*	3	4
*Pseudomonas* spp.^f^	3	3
*Granulicatella adiacens*	2	3
*Mycobacterium* spp.^g^	2	2
*Corynebacterium* spp.^h^	1	5
*Finegolida magna.*	1	2
*Serratia marcescens*	1	1
*Mycoplasma salivarium*	1	1
*Brucella melitensis*	1	1
*Enterobacter cloacae*	1	1
*Anaerococcus* spp.^i^	1	2
*Escherichia coli*	0	1
*Klebsiella pneumoniae*	0	1
*Rothia mucilaginosa*	0	1
*Parvimonas micra*	0	1
*Citrobacter braakii*	0	1
*Acinetobacter* johnsonii	0	1
*Human gammaherpes virus*	0	1

*The values are given as the detected frequency of a corresponding pathogen in all specimens. mNGS, Metagenomic next-generation sequencing.

a Including *S. epidermidis, S. haemolyticus, S. capitis, S. lugdunensis, S. hominis, S. lentus, and S. warneri*.

b Including *S. agalactiae, S. dysgalactiae, S. hemolyticus, S. mitis, S. sanguinis, S. gallolyticus, S. salivarius, S. gordonii, S. oralis*, and *S. tigurinis*.

c Including *Propionibacterium avidum* and *Cutibacterium acnes*.

d Including *Enterococcus faecalis* and *E. faecium*.

e Including *Candida albicans* and *Candida parapsilosis*.

f Including *Pseudomonas aeruginosa* and *Pseudomonas oleovorans*.

g Including *Mycobacterium bovis*, *Mycobacterium tuberculosis*, and *Mycobacterium abscessus*.

h Including *Corynebacterium striatum, Corynebacterium jeikeium, Corynebacterium amycolatum, Corynebacterium pseudogenitalium, Corynebacterium Kutscher*, and *Corynebacterium pyruviciproducens*.

i Including *Anaerococcus hydrogenalis* and *Anaerococcus prevotii*.

**Table 4:** Pathogens from partial concordant, discordant and negative-culture PJIs in the subset of patients with previous antibiotics therapy.

Case Classification	Missed Identifications by mNGS (No. of Cases)	New Identifications by mNGS (No. of Cases)
Positive-culture PJIs (N=58)	*Bacillus cereus* (3) *Cutibacterium acnes* (1) *Staphylococcus epidermidis* (1) *Staphylococcus caprae* (1) *Streptococcus mitis* (1) *Pseudomonas aeruginosa* (1) *Mycobacterium abscessus* (1)	*Staphylococcus aureus* (2) *Bacillus cereus* (2) *Streptococcus gordonii* (1) *Klebsiella pneumonia* (1) *Candida parapsilosis* (1) *Rothia mucilaginosa* (1)
Negative-culture PJIs (N=18)	NA	*Streptococcus agalactiae* (2) *Streptococcus hemolyticus* (1) *Streptococcus dysgalactiae* (1) *Staphylococcus aureus* (2) *Staphylococcus lentus* (1) *Staphylococcus epidermidis* (1) *Citrobacter braakii* + *Escherichia coli* + *Human gammaherpes virus* (1) *Candida parapsilosis* + *Candida albicans* (1) *Corynebacterium pseudogenitalium* (1) *Corynebacterium Kutscher* (1)

PJI, Periprosthetic joint infection; mNGS, Metagenomic next-generation sequencing.

Similarly, in cases without prior antimicrobial therapy, 16 cases (29.1%) tested positive for mNGS but negative for culture, 1 case (1.8%) tested negative for mNGS but positive for culture, 6 cases (10.9%) showed identical negative results, and 32 cases (58.2%) showed both positive culture and mNGS results. Of the 32 double-positive cases, there was complete concordance between culture and mNGS results in 24 cases (75.0%). Eight cases showed partial concordance (5 of 32 [15.6%]) or discordance (3 of 32 [9.4%]) of the microbial findings (**Table 5**).

**Table 5:** Pathogens from partial concordant, discordant and negative-culture PJIs in the subset of patients without previous antibiotics therapy.

Case Classification	Missed Identifications by mNGS (No. of Cases)	New Identifications by mNGS (No. of Cases)
Positive-culture PJIs (N=33)	*Cutibacterium acnes* (1) *Streptococcus viridans* + *Streptococcus mitis* (1) *Streptococcus mitis* (1) *Staphylococcus epidermidis* (1) *Pseudomonas aeruginosa* (1) *Bacillus cereus* (1)	*Anaerococcus johnsonii* + *Cutibacterium acnes* (1) *Parvimonas micra* (1) *Streptococcus oralis* (1) *Streptococcus sanguinis* (1) *Staphylococcus warneri* (1) *Staphylococcus haemolyticus* (1)
Negative-culture PJIs (N=22)	NA	*Cutibacterium acnes* (3) *Staphylococcus aureus* (1) *Staphylococcus lentus* (1) *Staphylococcus lugdunensis* (1) *Streptococcus agalactiae* (1) *Corynebacterium jeikeium* (1) *Corynebacterium pyruviciproducens* (1) *Pseudomonas aeruginosa* (1) *Pseudomonas oleovorans* (1) *Anaerococcus prevotii* (1) *Granulicatella adiacens* (1) *Mycobacterium tuberculosis* (1) *Finegoldia magna* (1) *Mycoplasma salivarium* (1)

PJI, Periprosthetic joint infection; mNGS, Metagenomic next-generation sequencing.

**Figure 5:**
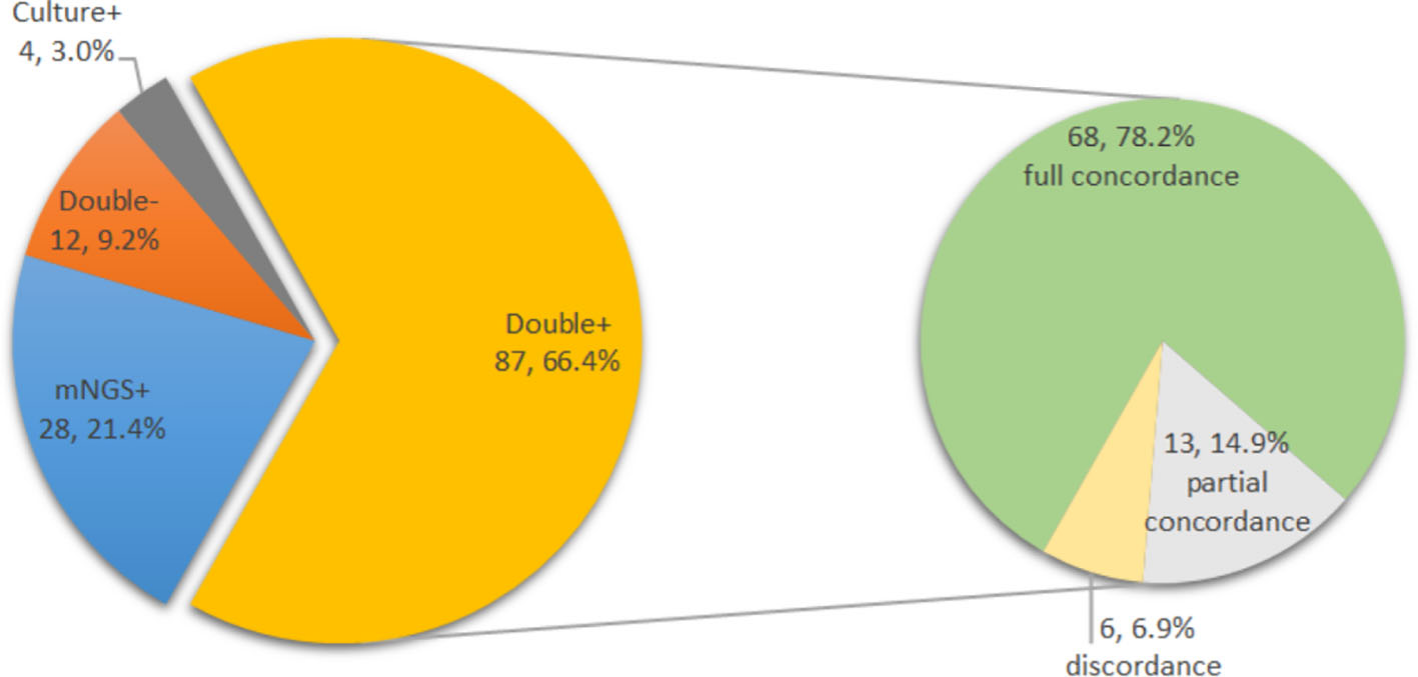
Distribution of mNGS and culture results in all cases (N=131) and concordance analysis between positive mNGS and positive culture cases (double positive, N=87).

The complete concordance of positive mNGS and positive culture results was similar in both groups (44 of 55 [80.0%] with previous antimicrobial therapy vs. 24 of 32 [75.0%] without previous antimicrobial therapy, p=0.586). The overall concordance analysis of all cultures from all sources and mNGS results is shown in **Figure 5**. For the double-positive subgroup, a high proportion of complete concordance (68 of 87, 78.2%) and partial concordance (13 of 87, 14.9%) was observed, with only six disagreements (6.9%) between mNGS and culture results. Of the six discordant cases, three were inconsistent at species level but consistent at genus level.

### Comparison of the cost and turnaround time between culture and mNGS

From sampling to reporting, the average days required for mNGS were 1.2 ± 0.4 and the average cost was 3234.6 ± 9.8 CNY. Meanwhile, the average turnaround time for culture results was 5.1 ± 2.6 days and the average cost was 696.8 ± 87.3 CNY. The turnaround time for obtaining the results of mNGS was significantly shorter than that of culture (t=9.688, p<0.001), and the cost of mNGS was significantly higher than that of culture (t=-241.3, p<0.001).

## Discussion

PJI is a catastrophic complication following knee or hip arthroplasty, and microbial culture is considered the gold standard for pathogen identification and diagnosis. However, due to preoperative antimicrobial therapy, the presence of biofilms and fastidious bacteria, the positivity rate has been low (Kheir et al., 2018; Li et al., 2019). Currently, diagnosis is still a challenge in the field of orthopaedics (Kapadia et al., 2016; Triantafyllopoulos et al., 2016). With the rapid development of culture-independent molecular technologies over the last decade, there have been increasing reports on the use of mNGS for pathogen identification, demonstrating that mNGS is superior to traditional culture in the diagnosis of PJI (He et al., 2021; Hao et al., 2023).

A total of 131 PJIs were included in this study. There were 115 patients with positive mNGS results and 91 patients with positive cultures. The positivity rate of mNGS in synovial fluid (87.8%) is significantly higher than in synovial fluid (50.4%) or deep tissue (61.8%) or the combination of two sources (69.5%). Compared with all cultures from all sources (synovial fluid and deep tissue), synovial fluid mNGS identified identical pathogens in 90.1% (82/91) of culture-positive PJIs, with additional potential pathogens detected in 15.4% (14/91). Meanwhile, new potential pathogens were detected in 70.0% (28/40) of culture-negative PJIs, demonstrating its usefulness in difficult-to-diagnose infections. In our institution, antibiotics were not commonly used in patients with suspected PJI prior to aetiological testing, and vancomycin was used empirically only when PJI was confirmed and the pathogen was unclear. However, 9/26 potential pathogens detected by mNGS in the 40 culture-negative PJIs were not covered by vancomycin. This may be one of the main reasons for the failure of anti-infective therapy. mNGS may have the potential to be a new powerful tool for the diagnosis of PJI. Thoendel et al. used metagenomic shotgun sequencing to detect a wide range of PJI pathogens from 408 cerebrospinal fluid samples and suggested that this method has the potential to improve or change the identification of many difficult-to-detect pathogens (Thoendel et al., 2018). Compared with microbial culture, mNGS identified identical pathogens in 94.8% of culture-positive PJIs and detected potential pathogens in 43.9% of culture-negative PJIs. Similarly, several other studies reported that the positive detection rate of mNGS was 83.0-97.1% in culture-positive PJIs (Ivy et al., 2018; Hao et al., 2023) and 25-81.8% in samples with negative culture results (Ivy et al., 2018; Tarabichi et al., 2018), indicating that this method can be used as an alternative to culture. The proportion of total and partial concordance in cases with concurrent positive culture and positive mNGS was 93.1% (81/87), which is an acceptable concordance rate. Furthermore, of the six discordant cases, three were discordant at species level but concordant at genus level.

It is well known that the detection of pathogens by culture may be impaired in PJIs previously treated with antibiotics (Trampuz et al., 2007). Antimicrobials should be discontinued 2 or even 4 weeks before sampling (Malekzadeh et al., 2010). However, it is not clear whether antimicrobials should be stopped before mNGS testing. To our knowledge, this is the first study to focus on the influence of prior antimicrobial therapy and different antimicrobial-free intervals on the positive detection rate of mNGS in synovial fluid in patients with PJI.

In this study, no significant differences were found in the positivity rates of deep tissue culture and synovial fluid mNGS between cases with and without prior antimicrobial therapy. However, the positivity rate of synovial fluid culture was higher in cases with previous antimicrobial therapy than in cases without antibiotics (57.9% vs 40.0%, p=0.043). It appears that the administration of antimicrobial therapy had no effect on the diagnostic microbial yield. These findings contrasted with those of previous studies confirming the influence of prior antimicrobial therapy on the detection rate (Malekzadeh et al., 2010; He et al., 2021). However, they were similar to those reported by Schulz et al. (2021). This may be the result of a selection bias, as previous antimicrobial therapy was more often seen in PJIs caused by acute infection, predominantly by highly virulent pathogens, and in these patients antimicrobial therapy has a weaker effect on culture positivity rate compared to chronic infection (**Figure 2**). This observation is supported by the fact that the positivity rate of non-microbiological diagnostic tests, especially the percentage of synovial PMN, was higher in the antimicrobial therapy group. In the meanwhile, the average number of reads for the top known pathogen identified in cases with and without previous antimicrobial therapy was comparable, as was that for acute PJIs in both groups. Although the average number of reads for the top known pathogen identified in chronic PJIs was significantly higher in cases without previous antimicrobial therapy than cases with previous antimicrobial therapy, mNGS showed mNGS showed no significant difference in the positivity rate of acute and chronic PJIs in both groups.

In this study, the average number of days required for culture was 5.1 days and 1.2 days for mNGS. Although the cost of mNGS was higher than that of culture, considering the higher positivity rate and earlier resolution capability, mNGS was more beneficial for early targeted antibiotic therapy for PJI. According to a study by Torchia et al. (2019), mNGS was more cost-effective than culture and should therefore be used as the standard method for PJI in clinical practice.

Another interesting finding in this study was that the trends in culture and mNGS positivity rates with different antimicrobial-free intervals. The positivity rates of deep tissue culture and synovial fluid culture all decreased with shortening of the antimicrobial-free interval (>14 days, 4 to 14 days, 0 to 3 days) in patients receiving antimicrobial therapy prior to sampling. For synovial fluid mNGS, although the positivity rate did not show a consistent downward trend, it did decrease as the antimicrobial-free interval increased from 4 to 14 days to 0 to 3 days. This may mean that antimicrobials should be discontinued more than 3 days before sampling to further increase the positivity rate of mNGS for PJI.

There are several limitations to the present study, including the fact that no gold standard has yet been established for the diagnosis of pathogens in PJI. Contaminants would then be a potential interference for mNGS and culture, which could influence the analysis. The low incidence of PJI limited the sample size, which may reduce the accuracy of some statistical results in this study. The high cost of mNGS limited the use of mNGS in deep tissue samples to fully evaluate the influence of prior antimicrobial therapy on the detection rate. The type and duration of previous antimicrobials were not further analysed. In addition, this was a single-centre study, which may be subject to some bias. A multicentre study with a larger sample size should be carried out next.

## Conclusion

In conclusion, mNGS had a significantly higher positivity rate than culture in detecting PJI, suggesting that mNGS may be a promising tool for rapid aetiological diagnosis of PJI, especially in PJI patients with a negative culture result or history of antibiotic administration. However, our data suggest that antimicrobials should be discontinued more than 3 days prior to sampling to further increase the positivity rate of mNGS for PJI.

## Data availability statement

The datasets presented in this study can be found in online repositories. The names of the repository/repositories and accession number(s) can be found below: https://www.ncbi.nlm.nih.gov/, PRJNA877476.

## Ethics statement

The studies involving humans were approved by the Ethics Committee of Honghui Hospital Affiliated to Xi’an Jiao Tong University. The studies were conducted in accordance with the local legislation and institutional requirements. The participants provided their written informed consent to participate in this study.

## Author contributions

LH: Data curation, Funding acquisition, Project administration, Writing – original draft, Writing – review & editing. WB: Writing – review & editing. ZQ: Writing – review & editing. TM: Writing – review & editing. PX: Project administration, Supervision, Writing – review & editing. HL: Project administration, Supervision, Writing – review & editing. PW: Funding acquisition, Writing – review & editing.
