# Supplementary material for: Will previous antimicrobial therapy reduce the positivity rate of metagenomic next-generation sequencing in periprosthetic joint infections? A clinical study
Source: Front Cell Infect Microbiol. 2024 Jan 11;13:1295962. doi: 10.3389/fcimb.2023.1295962 (PMC10808557; doi:10.3389/fcimb.2023.1295962)
Supplement: Supplementary file 1 [file DataSheet_1.pdf]

Supplementary Methods

mNGS Detection

The workflow of mNGS in synovial fluid included sample preparation, nucleic acid extraction, construction of DNA libraries, metagenomic sequencing and bioinformatics analysis.

Sample preparation and DNA exaction

Upon arrival at the sequencing laboratory, 1.5-mL microcentrifuge tubes with 0.5 mL of sample and 1 g of 0.5-mm glass beads were attached to a horizontal platform on a vortex mixer and agitated vigorously at 2800–3200 rpm for 30 min. After agitation, 0.3 mL of the sample was separated into a new 1.5-mL microcentrifuge tube, and DNA extraction was performed using the TIANamp Micro DNA Kit (DP316, Tiangen Biotech, China) according to the manufacturer's recommendation. Add Proteinase K to the microcentrifuge tube and mix thoroughly. Then add Buffer GB to the sample, mix thoroughly, and incubate at 56 ℃ for 10 min to yie ld a homogeneous solution. Briefly centrifuge the 1.5 ml microcentrifuge tube to remove drops from the inside of the lid. Add ethanol (96-100%) to the sample. Mix thoroughly and Incubate at room temperature for 5 minutes. Briefly centrifuge the 1.5 ml microcentrifuge tube to remove drops from the inside of the lid. Pipet the mixture from step 4 into the Spin Column CR2 (in a 2 ml Collection Tube) and centrifuge at 12,000 rpm (~13,400 × g) for 30 sec. Discard flow-through and place the spin column into the Collection Tube. Add Buffer GD (Ensure ethanol has been added) to Spin Column CR2, and centrifuge at 12,000 rpm (~13,400 × g) for 30 sec. Then discard the flow-through and place the spin column into the Collection Tube. Add Buffer PW (Ensure ethanol has been added) to Spin Column CR2, and centrifuge at 12,000 rpm (~13,400 ×g) for 30 sec. Discard the flow-through and place the spin column into the Collection Tube. Add Buffer PW (Ensure ethanol has been added) to Spin Column CR2 again, and centrifuge at 12,000 rpm (~13,400 × g) for 30 sec. Discard the flow-through and place the spin column into the Collection Tube. Centrifuge at 12,000 rpm (~13,400 × g) for 2 min to dry the membrane completely. Place the Spin Column CR2 in a new clean 1.5 ml microcentrifuge tube, and pipet Buffer TB directly to the center of the membrane. Incubate at room temperature for 2-5 min, and then centrifuge for 2 min at 12,000 rpm (~13,400 × g). The extracted DNA should be stored at -20 ℃.

Construction of DNA libraries

The construction of DNA library was prepared using the MGIEasy FS DNA Library Prep Kit (MGI Tech., China) through an end-repair method. By randomly breaking template DNA into fragments (200~300bp) through Covaris S220 (Thermo Fisher Science, USA) ultrasonic crusher. End Repair and A-tailing were performed simultaneously. Add MGIEasy DNA Adapters and purify the adapter-ligated DNA with beads. After polymerase chain reaction (PCR) amplification was performed, purification of the PCR product was conducted again. The quality of the DNA libraries was assessed using an Agilent

2100 Bioanalyzer (Agilent Technologies, Santa Clara, California) combined with quantitative PCR to measure the adapters before sequencing.

Metagenomic sequencing and bioinformatics analysis

The qualified DNA library was denatured and cyclified to form a single strand circularization. After the process of rolling circle amplification, DNA nanoball was prepared and loaded onto the Patterned Array. Sequencing was performed using the BGISEQ-500 platform. A negative control and a positive control of a known pathogen were set up for the same batch of samples. If obvious contamination was found, the specimen was retested again. The bioinformatics analysis included the following main steps: 1) High-quality sequencing data were generated by removing short (shorter than 35 bp), low-quality, low-complexity reads, adapter contamination, and duplicated reads. 2) Human host sequences were eliminated by mapping to the human reference genome (hg19) with Burrows-Wheeler Alignment (http://bio-bwa.sourceforge.net). 3) The remaining sequencing data were aligned to the current bacterial, virus, and fungal databases (ftp://ftp.ncbi.nlm.nih.gov/genomes/), which contain the genomic sequences of 4,061 viruses, 2,473 bacteria and 199 fungi related to human diseases. The reference genomes in the database were downloaded from the National Center for Biotechnology Information (NCBI). The contamination was eliminated by referring to the same batch of negative control. Reference genome coverage was also used to help distinguish background contaminant reads from bacteria present in the sample. Pathogens were ranked according to the read counts, genome coverage rate and species abundance and screened according to detection threshold.

Interpretation of mNGS results (Figure 1)

The sequencing algorithm we adopted in this study with reference to the analysis method by Ivy MI et al[1].

1. Bacteria (except Mycobacterium)

Organisms with a coverage rate 10 times or more than the other organisms were considered to be the pathogenic species. For an organism that did not match the negative control pathogen, it was considered to be the pathogenic species if the number of reads stringently mapped to the pathogen at the genus level was ≥10. For an organism that matched the negative control pathogen, it was considered positive if the coverage rate was ≥2% and the number of reads stringently mapped to pathogen at the genus level was ≥10 in two consecutive tests.

2. Fungi and virus

Due to low nucleic acid yield, if the number of reads mapped to pathogen at genus or species level ≥10 and in the top 10 for bacteria, they were considered positive.

3. Mycobacterium:

Due to the extremely low nucleic acid yield, if the number of reads stringently mapped to pathogen at genus level ≥1 and the number was in the top 10, it was considered to be the pathogenic species.

4. Parasites were not considered to be pathogens in general.

5. *Burkholderia, Ralstonia, Delftia, Sphingobium, Alternaria, Sodaria, Aspergillus, Albugo*, and other genera were the most common background bacteria and were also detected in other sample types in our laboratory.

6. For cases with negative microbial culture but positive mNGS results and cases with microbial culture suggesting monobacterial infections but mNGS suggesting multiple infections and cases with complete inconsistent results obtained from culture and mNGS, three criteria were used to determine whether the mNGS results were "true positives" based on previous literature[2,3]: (1) using a third method, the results were consistent with the results of mNGS, such as 16S PCR. (2) Pathogens were clearly reported in osteoarticular infection, according to previous studies, which was consistent with the clinical characteristics of the patient. (3) Targeted treatment response determined by at least three senior clinicians.

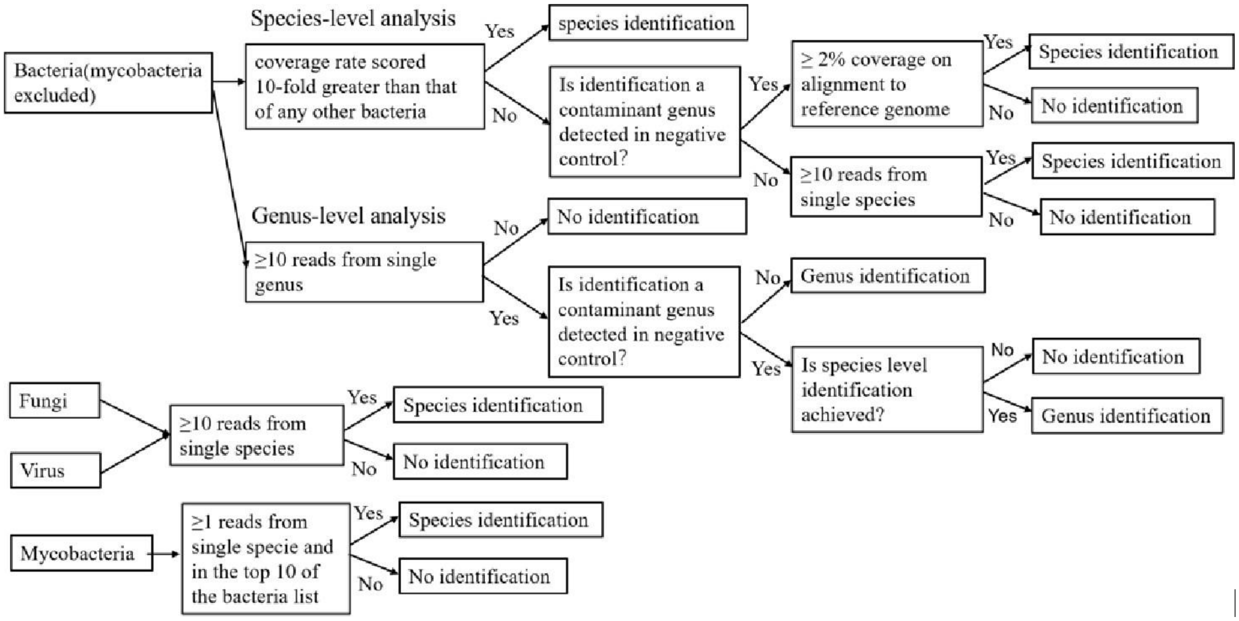

Figure 1. The identification algorithm of a positive mNGS results

References

1. Ivy MI, Thoendel MJ, Jeraldo PR, Greenwood-Quaintance KE, Hanssen AD, Abdel MP, et al. Direct Detection and Identification of Prosthetic Joint Infection Pathogens in Synovial Fluid by Metagenomic Shotgun Sequencing. J Clin Microbiol. (2018) 56(9):e00402-18. doi:10.1128/JCM.00402-18

2. Wang J, Han Y, Feng J. Metagenomic next-generation sequencing for mixed pulmonary infection diagnosis. BMC Pulm Med. (2019) 19(1):252. doi:10.1186/s12890-019-1022-4

3. Xie G, Zhao B, Wang X, Bao L, Xu Y, Ren X, et al. Exploring the Clinical Utility of Metagenomic Next-Generation Sequencing in the Diagnosis of Pulmonary Infection. Infect Dis Ther. (2021) 10(3):1419-35. doi:10.1007/s40121-021-00476-w
