# Supplementary material for: Will previous antimicrobial therapy reduce the positivity rate of metagenomic next-generation sequencing in periprosthetic joint infections? A clinical study
Source: Front Cell Infect Microbiol. 2024 Jan 11;13:1295962. doi: 10.3389/fcimb.2023.1295962 (PMC10808557; doi:10.3389/fcimb.2023.1295962)
Supplement: Supplementary file 2 [file DataSheet_2.pdf]

# Non- and microbiologic results of cases with previous antimicrobial therapy

| **Sample Number** | **Intraoperative Deep-tissue Culture Results** (# positive/total # | **Intraoperative Synovial Fluid Culture Results** (0 = no growth) | **Total Paired Reads Sequenced** | **mNGS Results** | **Pathogen sequencing reads** | **Preoperative Antibiotics within 4** | **Antibiotics free interval**(1=0~3d, 2=4~1 | **Preoperative Antibiotic Name** | **Age at extraction** (years) | **Sex** | **Side** (right or left) | **Joint** (K=knee H=hip) | **Acute Infection** (0=neg, 1=pos) | **Intraoperative Purulence** (0=no, 1=yes, 2=surge | **Sinus Tract** (0=no, 1=yes, nk=not known) | **WBC** ($10^3$ cells/mm$^3$, nd=not done) | **Serum ESR** (mm/h, nd=not done, Ref range: | **Serum CRP** (mg/L, nd=not done, Ref | **Total nucleated cell count** (cells/mm$^3$ | **% Neutrophils** | **Histopathology** (0=neg, 1=pos, nd=not | **Histopathology description** |
|---|---|---|---|---|---|---|---|---|---|---|---|---|---|---|---|---|---|---|---|---|---|---|
| 1 | Viridans group Streptococcus 4/4 | Viridans group Streptococcus | 35437545 | Viridans group Streptococcus (*S. tigurinis*) | 146 | 1 | 3 | Daptomycin, cefepime | 68 | M | L | H | 1 | 1 | 0 | 20 | 19 | 41 | nd | nd | 1 | Acutely inflamed granulation tissue with acute purulent exudate |
| 2 | Pseudomonas aeuruginosa 3/3 | Pseudomonas aeruginosa | 30384567 | Pseudomonas aeruginosa | 234 | 1 | 3 | Cefepime | 82 | M | R | K | 1 | 1 | 0 | 8 | 68 | 108 | 37760 | 83 | 1 | Arthroplasty effect with fibrin and acute and chronic inflammation |
| 3 | Coagulase-negative Staphylococcus 2/3 | 0 | 33468556 | *Staphylococcus epidermidis* | 54 | 1 | 3 | Unknown | 71 | F | R | K | 0 | 0 | 0 | 6.5 | 84 | 20.7 | nd | nd | 1 | Fibrovascular tissue with acute and chronic inflammation |
| 4 | S. aureus 4/4 | S. aureus | 34989427 | *Staphylococcus aureus* | 583 | 1 | 2 | Trimethoprim/sulfamethoxazole | 78 | F | R | K | 1 | 1 | 0 | 5.4 | 56 | 38.6 | nd | nd | 1 | Reactive synovial tissue with focal acute inflammation |
| 5 | 0/3 | 0 | 32421234 | Negative | 142 | 1 | 1 | Rifampicin, Levofloxacin | 81 | F | L | K | 1 | 0 | 0 | 5.9 | 24 | 29.8 | 130200 | 93 | 0 | Arthroplasty effect, negative for acute inflammation |
| 6 | Coagulase-negative Staphylococcus 3/4 | Coagulase-negative Staphylococcus | 33262575 | *Staphylococcus epidermidis* | 2043 | 1 | 2 | Vancomycin, norfloxacin | 54 | M | R | K | 1 | 1 | 0 | 9.7 | 25 | 14.9 | 75095 | 88 | 1 | Arthroplasty effect and inflamed granulation tissue with moderate acute inflammation |

| | | | | | | | | | | | | | | | | | | | | | |
|---|---|---|---|---|---|---|---|---|---|---|---|---|---|---|---|---|---|---|---|---|---|
| 7 | Enterococcus faecalis 2/3 | Enterococcus faecalis | 29347399 | *Enterococcus faecalis* | 96 | 1 | 2 | Amoxicillin | 74 | F | R | K | 1 | 1 | 1 | 6.5 | 22 | 19.3 | 9135 | 89 | 0 | Arthroplasty effect, negative for acute inflammation |
| 8 | Coagulase-negative Staphylococcus 2/3 | Coagulase-negative Staphylococcus | 30296730 | *Staphylococcus epidermidis* | 394 | 1 | 3 | Amoxicillin, clindamycin | 51 | M | R | H | 1 | 1 | 1 | 8.8 | 24 | 14 | nd | nd | 1 | Dense fibrous tissue with acute inflammation |
| 9 | 0/3 | Enterobacter cloacae | 29833936 | *Enterobacter cloaecae* | 82 | 1 | 3 | Vancomycin, meropenem | 61 | M | R | H | 1 | 0 | 1 | 10 | 33 | 39.6 | 70397 | 97 | 1 | Arthroplasty effect with acute inflammation |
| 10 | Coagulase-negative Staphylococcus 3/4，C. acnes 1/4 | Coagulase-negative Staphylococcus | 31950316 | *Staphylococcus epidermidis* | 107 | 1 | 3 | Daptomycin | 54 | F | L | H | 1 | 0 | 0 | 11 | 25 | 19.5 | 30785 | 88 | 1 | Acute and chronic inflammation and arthroplasty effect |
| 11 | 0/4 | Coagulase-negative Staphylococcus | 31880973 | *Staphylococcus epidermidis* | 43 | 1 | 3 | Minocycline | 83 | F | R | K | 0 | 1 | 0 | 6.5 | 25 | 3.4 | 629 | 51 | nd | |
| 12 | Bacillus sp. 1/3 | 0 | 35351619 | staphylococcus aureus | 115 | 1 | 1 | Ceftriaxone, levofloxacin, penicillin | 64 | F | R | H | 1 | 0 | 0 | 7 | 108 | 380.2 | 43764 | 98 | 1 | Synovial tissue with acute inflammation |
| 13 | Coagulase-negative Staphylococcus 1/4 | S. epidermidis | 31001942 | *Staphylococcus epidermidis* | 75 | 1 | 1 | Cefadroxil, daptomycin | 47 | M | R | H | 0 | 1 | 0 | 8.3 | 35 | 52.3 | 58464 | 97 | 1 | Fibrous tissue with focal acute inflammation |

| | | | | | | | | | | | | | | | | | | | | | | |
|---|---|---|---|---|---|---|---|---|---|---|---|---|---|---|---|---|---|---|---|---|---|---|
| 14 | 0/4 | 0 | 31970842 | *Streptococcus agalactiae* | 77 | 1 | 1 | Ceftriaxone | 60 | M | R | K | 1 | 0 | 0 | 6.5 | 45 | 32.2 | 10586 | 96 | 0 | Granulation tissues and fibrinous debris |
| 15 | 0/3 | Streptococcus agalactiae | 32231355 | *Streptococcus agalactiae* | 125 | 1 | 2 | Cefadroxil | 62 | M | R | K | 0 | 1 | 0 | 12 | 97 | 38.5 | 35826 | 91 | 1 | Acute necroinflammatory debris |
| 16 | 0/4 | 0 | 25197516 | *Citrobacter braakii, Escherichia coli,Human gammaherpes virus* | 51 | 1 | 2 | Cephalexin | 84 | F | R | K | 0 | 1 | 0 | 13.8 | 31 | 9.4 | 1556 | 89 | nd | |
| 17 | 0/3 | 0 | 25462106 | staphylococcus lentus | 12 | 1 | 1 | Trimethoprim/sulfamethoxazole | 67 | M | R | H | 1 | 1 | 0 | 8.3 | 9 | 39.3 | nd | nd | nd | |
| 18 | Streptococcus bovis gp. 4/4 | Streptococcus bovis gp. | 32497858 | *Streptococcus gallolyticus (bovis gp)* | 90 | 1 | 3 | Cefazolin | 72 | M | L | H | 1 | 2 | 0 | 11.6 | 43 | 67 | 64001 | 75 | nd | |
| 19 | S. epidermidis 3/4 | 0 | 31402589 | *Staphylococcus epidermidis* | 94 | 1 | 1 | Ertapenem | 58 | M | R | H | 0 | 1 | 1 | 8.1 | 25 | 6.8 | 52 | 83 | 0 | Arthroplasty effect, with granulation tissue, fibrosis, and reactive bone |
| 20 | 0/3 | 0 | 27736224 | *Corynebacterium pseudogenitalium* | 86 | 1 | 1 | Azithromycin, Ciprofloxacin, Meropenem, Vancomyci | 74 | M | R | K | 1 | 1 | 0 | 11.8 | 47 | 239.9 | 42681 | 92 | nd | |

| | | | | | | | | | | | | | | | | | | | | | | |
|---|---|---|---|---|---|---|---|---|---|---|---|---|---|---|---|---|---|---|---|---|---|---|
| 21 | S. epidermidis 1/3 | 0 | 31314360 | Negative | | 1 | 3 | Unknown | 65 | F | L | K | 0 | 0 | 0 | 6 | 8 | 2 | 15169 | 78 | 1 | Granulation tissue with chronic inflammation |
| 22 | 0/5 | 0 | 31070514 | Negative | | 1 | 1 | Ciprofloxacin | 51 | F | R | K | 0 | 0 | 1 | 6.3 | 19 | 6.5 | 1230 | 82 | 1 | Prior arthroplasty with patchy acute inflammation |
| 23 | Bacillus sp. 1/5 | 0 | 31959946 | Negative | | 1 | 2 | Nafcillin, rifampin | 63 | F | L | H | 0 | 2 | 0 | 5.4 | 95 | 12.8 | nd | nd | 1 | Synovial tissue with acute inflammation |
| 24 | S. epidermidis 3/3 | Staphylococcus epidermidis | 31243164 | *Staphylococcus epidermidis* | 409 | 1 | 2 | Amoxicillin/ clavulanic acid | 71 | M | L | K | 1 | 1 | 1 | 7.6 | 52 | 84.9 | 41613 | 98 | nd | |
| 25 | 0/5 | 0 | 27054741 | *Staphylococcus epidermidis* | 42 | 1 | 2 | Ciprofloxacin | 68 | F | R | K | 0 | 2 | 0 | 10.6 | 17 | 31.3 | 90005 | 78 | 1 | Significant acute inflammation present |
| 26 | 0/3 | Granulicatella adiacens | 30889357 | *Granulicatella adiacens* | 27 | 1 | 2 | Cephalexin | 63 | F | L | K | 0 | 1 | 0 | 40.6 | 31 | 130.8 | 24215 | 86 | 1 | Granulation tissue with acute inflammation |
| 27 | Serratia sp 5/5, S. epidermidis 1/5 | Serratia sp. | 26176872 | *Serratia marcescens. Staphylococcus epidermidis* | 160 | 1 | 2 | Clindamycin | 45 | M | R | K | 1 | 1 | 1 | 7.8 | 71 | 48.2 | nd | nd | 1 | Acute inflammation |

| | | | | | | | | | | | | | | | | | | | | | | |
|---|---|---|---|---|---|---|---|---|---|---|---|---|---|---|---|---|---|---|---|---|---|---|
| 28 | 0/3 | 0 | 40652623 | Negative | | 1 | 1 | Vancomycin, cefazolin | 83 | M | L | K | 0 | 1 | 0 | 7.6 | 81 | 145.7 | 32970 | 90 | 1 | Granulation tissue with acute inflammation |
| 29 | C. albicans 3/5, S. epidermidis 1/5 | 0 | 33098682 | C. albicans, S. epidermidis, Bacillus sp. | 99 | 1 | 2 | Cefadroxil | 61 | M | L | H | 0 | 2 | 1 | 6.7 | 6 | 3 | nd | nd | 0 | Arthroplasty effect with rare neutrophils present |
| 30 | 0/3 | S. aureus | 33448840 | *Staphylococcus aureus* | 1113 | 1 | 2 | Unknown, cefazolin | 59 | M | R | H | 1 | 2 | 0 | 7.4 | 52 | 132.9 | 115241 | 81 | 1 | Fibrous tissue with acute inflammation |
| 31 | S. epidermidis 3/4 | S. epidermidis | 30153552 | S. epidermidis，Klebsiella pneumoniae | 87 | 1 | 3 | Minocycline | 76 | M | R | K | 0 | 1 | 0 | 9 | 43 | 25 | 13959 | 74 | 1 | Arthroplasty effect effect with acute inflammation |
| 32 | 0/5 | 0 | 34158596 | Streptococcus hemolyticus | 549 | 1 | 1 | Unknown | 45 | M | R | K | 0 | 1 | 0 | 29.4 | 36 | 208.4 | 19920 | 99 | 1 | Acute inflammation and necrotic tissue |
| 33 | S. epidermidis 1/6, S. caprae 1/6 | S. epidermidis | 28649863 | *Staphylococcus epidermidis* | 1940 | 1 | 1 | Unknown | 73 | F | R | K | 1 | 1 | 0 | 7.9 | 55 | 18.3 | nd | nd | 1 | Synovium with granulation tissue and acute inflammation |
| 34 | S. hominis 1/3 | S. hominis | 29484544 | S. hominis | 43 | 1 | 1 | Unknown, vancomycin, cefepime | 66 | M | L | H | 0 | 0 | 1 | 6.8 | 85 | 111 | 886 | 2 | 1 | Fibroconnective tissue with acute inflammation |

| | | | | | | | | | | | | | | | | | | | | | | |
|---|---|---|---|---|---|---|---|---|---|---|---|---|---|---|---|---|---|---|---|---|---|---|
| 35 | P. aeruginosa 1/3 | P. aeruginosa | 28054688 | *Staphylococcus aureus* | 467 | 1 | 2 | Vancomycin, cefepime, cefazolin, trimethoprim/sulfamethoxazole | 76 | M | R | H | 1 | 1 | 0 | 10.5 | 55 | 32.1 | 18480 | 77 | 1 | Arthroplasty effect with mild acute inflammation |
| 36 | E. faecium 1/3 | E. faecium | 27958179 | *Enterococcus faecium* | 10867 | 1 | 2 | Minocycline | 68 | M | R | K | 1 | 1 | 0 | 17.5 | 53 | 59.3 | 32708 | 85 | nd | |
| 37 | 0/3 | 0 | 28255833 | *Streptococcus dysgalactiae* | 3769 | 1 | 1 | Ceftriaxone | 72 | M | R | K | 0 | 0 | 1 | 3.9 | 50 | 29.7 | 14377 | 95 | 1 | Granulation tissues and fibrinous debris with focal acute inflammation |
| 38 | S. epidermidis 3/6 | S. epidermidis | 29833639 | *Staphylococcus epidermidis* | 71 | 1 | 2 | Trimethoprim/sulfamethoxazole | 73 | M | L | K | 0 | 1 | 0 | 7.1 | 40 | 9.9 | nd | nd | 0 | Arthroplasty effect, negative for acute inflammation, perivascular chornic inflammation |
| 39 | S. epidermidis 1/3 | S. epidermidis | 27279393 | *Staphylococcus epidermidis* | 114 | 1 | 1 | Doxycycline | 78 | M | L | K | 1 | 1 | 0 | 5.9 | 31 | 27.7 | 16230 | 94 | 1 | Granulation tissue with chronic inflammation |
| 40 | 0/3 | Streptococcus agalactiae | 21709611 | *Streptococcus agalactiae. Candida parapsilosis* | 266 | 1 | 2 | Vancomycin, cephalexin | 56 | F | L | K | 0 | 0 | 0 | 8.1 | 78 | 399 | 61155 | 93 | nd | |
| 41 | S. epidermidis 1/4 | S. epidermidis | 27441490 | *Staphylococcus epidermidis* | 381 | 1 | 3 | Doxycycline | 72 | F | L | H | 1 | 2 | 0 | 4.6 | 67 | 51.9 | 44163 | 83 | 1 | Synovial tissue with acute inflammation |

| | | | | | | | | | | | | | | | | | | | | | |
|---|---|---|---|---|---|---|---|---|---|---|---|---|---|---|---|---|---|---|---|---|---|
| 42 | 0/4 | 0 | 21140017 | *Staphylococcus aureus* | 85 | 1 | 2 | Doxycycline | 68 | F | L | K | 0 | 2 | 0 | 10.3 | 36 | 37.4 | 19686 | 84 | 1 | Fibrous tissue with acute inflammation |
| 43 | S. epidermidis 1/3 | S. epidermidis | 21316638 | *Staphylococcus epidermidis* | 296 | 1 | 2 | Cefadroxil | 60 | M | R | H | 1 | 1 | 0 | 12.7 | 50 | 51.1 | 19194 | 89 | 1 | Fibrous tissue with acute inflammation |
| 44 | E. faecalis 3/4 | E. faecalis | 18183009 | *Enterococcus faecalis* | 94 | 1 | 1 | Trimethoprim/sulfamethoxazole, cephalexin | 50 | F | L | K | 1 | 0 | 0 | 6.2 | 55 | 52.7 | 64493 | 95 | 1 | Acute inflammation |
| 45 | Streptococcus mitis sp group 1/4 | 0 | 26592067 | Streptococcus mitis sp group，Rothia mucilaginosa | 116 | 1 | 1 | Vancomycin | 80 | F | R | K | 0 | 1 | 0 | 12.7 | 36 | 36 | 16192 | 95 | 1 | Fibrous tissue with acute inflammation |
| 46 | Enterococcus faecalis 1/4 | 0 | 32729575 | Enterococcus faecalis | 19 | 1 | 2 | Cefuroxime, levofloxacin, | 71 | M | R | K | 0 | 1 | 0 | 8.6 | 12 | 24 | 6637 | 57 | 1 | Granulation tissues and fibrinous debris with focal acute inflammation |
| 47 | S. epidermidis 2/3 | S. epidermidis | 24996259 | *Staphylococcus epidermidis* | 1406 | 1 | 1 | Trimethoprim/sulfamethoxazole | 60 | M | L | K | 1 | 1 | 0 | 6.2 | 8 | 16.9 | 18585 | 83 | 1 | Marked acute and chronic inflammation and granulation tissue |
| 48 | 0/4 | 0 | 31616449 | Negative | | 1 | 1 | Amoxicillin, trimethoprim/sulfamethoxazole | 56 | M | R | K | 0 | 2 | 0 | 9.6 | 25 | 4.6 | 779 | 20 | 1 | Granulation tissue with chronic inflammation and fibrin |

| | | | | | | | | | | | | | | | | | | | | | |
|---|---|---|---|---|---|---|---|---|---|---|---|---|---|---|---|---|---|---|---|---|---|
| 49 | S. aureus 1/5 | 0 | 26835306 | *Staphylococcus aureus* | 59 | 1 | 3 | Cefadroxil | 55 | F | R | K | 0 | 0 | 0 | 3.5 | 60 | 11.5 | 4004 | 83 | 1 | Arthroplasty effect with fibrin and chronic inflammation |
| 50 | S. epidermidis 3/3 | Coagulase-negative Staphylococcus | 28709020 | *Staphylococcus epidermidis* | 311 | 1 | 2 | Minocycline, vancomycin | 56 | M | R | K | 1 | 1 | 0 | 9.2 | 91 | 66.5 | 45463 | 86 | 1 | Acutely inflamed granulation tissue with acute purulent exudate |
| 51 | Corynebacterium striatum 3/3 | C. striatum | 32165206 | *Corynebacterium striatum* | 462 | 1 | 3 | Minocycline | 58 | F | R | K | 1 | 1 | 0 | 7.7 | 90 | 26.5 | 165913 | 90 | 1 | Acute inflammation |
| 52 | 0/3 | 0 | 23812627 | *Streptococcus agalactiae* | 81 | 1 | 1 | Vancomycin | 61 | M | R | K | 0 | 1 | 0 | 9.7 | 114 | 334.7 | 370771 | 89 | 1 | Granulation tissue with acute inflammation |
| 53 | 0/7 | Brucella melitensis | 31498435 | *Brucella melitensis* | 803 | 1 | 1 | Ertapenem | 62 | M | L | K | 1 | 1 | 0 | 9.7 | 89 | 186.7 | 25513 | 96 | 1 | Synovial tissue with acute inflammation |
| 54 | 0/5 | S. haemolyticus | 31323631 | *Staphylococcus haemolyticus.* | 134 | 1 | 3 | Vancomycin, rifampin, cefadroxil, ciprofloxacin | 69 | M | L | K | 0 | 2 | 1 | 7.4 | 22 | 57.2 | 20340 | 98 | 1 | Infection characterized by abscess associated with arthroplasty effect |
| 55 | S. epidermidis 3/4 . | S. epidermidis | 31652132 | *Staphylococcus epidermidis ， Bacillus sp.* | 289 | 1 | 2 | Trimethoprim/sulfamethoxazole | 59 | F | R | H | 1 | 1 | 0 | 8.8 | 51 | 74.6 | 16297 | 93 | 1 | Benign fibrovascular tissue with focal acute inflammation |

| | | | | | | | | | | | | | | | | | | | | | | |
|---|---|---|---|---|---|---|---|---|---|---|---|---|---|---|---|---|---|---|---|---|---|---|
| 56 | Enterococcus faecalis 3/4 | Enterococcus faecalis | 30145056 | *Enterococcus faecalis* | 55 | 1 | 3 | Meropenem, vancomycin | 60 | F | R | K | 1 | 0 | 0 | 7.7 | 130 | 78.5 | 52316 | 71 | 1 | Acute inflammation present |
| 57 | *S. lugdunensis 4/6* | *S. lugdunensis* | *3E+07* | *Staphylococcus lugdunensis* | 14 | 1 | 2 | Amoxicillin | 67 | M | L | H | 1 | 1 | 0 | 4.9 | 41 | 52.6 | nd | nd | 1 | Synovial tissue with acute inflammation |
| 58 | *S. epidermidis 2/3* | *S. epidermidis* | *2.6E+07* | *Staphylococcus epidermidis* | 29 | 1 | 3 | Minocycline | 67 | M | R | K | 1 | 1 | 0 | 5.9 | 30 | 40.9 | nd | nd | 1 | Granulation tissue with acute and chronic inflammation |
| 59 | M. abcessus group 2/3 | M. abscessus group | 15345964 | Negative | | 1 | 1 | Cefoxitin, azithromycin | 64 | M | R | K | 0 | 2 | 0 | 8.6 | 92 | 26.9 | 9915 | 92 | 1 | Granulation tissue with acute inflammation |
| 60 | C. acnes 1/5 | 0 | 29198727 | *C. acnes* | 17 | 1 | 2 | Minocycline | 68 | M | R | K | 0 | 1 | 0 | 7.4 | 35 | 14.8 | 3626 | 77 | 1 | Granulation tissue with chronic inflammation and fibrin |
| 61 | C. acnes 1/7 | 0 | 31413355 | *Cutibacterium acnes* | 159 | 1 | 3 | Minocycline | 83 | M | L | H | 0 | 1 | 0 | 7.8 | 34 | 21.9 | 3224 | 85 | 1 | Granulation tissue with chronic inflammation |
| 62 | S. aureus 3/5 | S. aureus | 27061830 | *Staphylococcus aureus* | 223 | 1 | 3 | Vancomycin, cefepime, daptomycin | 56 | F | R | H | 0 | 1 | 0 | 9.6 | 122 | 78 | 15794 | 96 | 1 | Acute inflammation present |

| | | | | | | | | | | | | | | | | | | | | | | |
|---|---|---|---|---|---|---|---|---|---|---|---|---|---|---|---|---|---|---|---|---|---|---|
| 63 | S. aureus 4/4 | 0 | 26343324 | *Staphylococcus aureus* | 89 | 1 | 1 | Minocycline | 67 | F | R | K | 1 | 1 | 0 | 10.2 | 78 | 48.1 | 7855 | 84 | 1 | Granulation tissues and fibrinous debris with focal acute inflammation |
| 64 | 0/3 | 0 | 27038349 | *Corynebacterium kutscher* | 19 | 1 | 2 | Doxycycline | 70 | M | L | K | 0 | 2 | 0 | 9.3 | 10 | 7.5 | 25561 | 93 | 1 | Synovium with acute inflammation |
| 65 | 0/3 | 0 | 27474718 | *Candida parapsilosis + Candida albicans* | 21 | 1 | 1 | Doxycycline | 70 | M | R | K | 0 | 2 | 0 | 9.3 | 10 | 7.5 | 7480 | 43 | 1 | Granulation tissue with chronic inflammation |
| 66 | S. aureus 3/3 | S. aureus | 36511359 | *Staphylococcus aureus* | 59 | 1 | 2 | Vancomycin | 56 | M | R | H | 0 | 2 | 0 | 16 | 15 | 189.1 | 104489 | 99 | 1 | Granulation tissue with acute inflammation |
| 67 | S. epidermidis 1/4 | 0 | 32378140 | *Staphylococcus epidermidis* | 503 | 1 | 2 | Cephalexin | 64 | F | L | K | 0 | 2 | 0 | 8.6 | 44 | 16.5 | 1769 | 60 | 1 | Soft tissue with chronic inflammation, no acute inflammation seen |
| 68 | S. epidermidis 2/4 | S. epidermidis | 26255107 | *Staphylococcus epidermidis* | 28 | 1 | 3 | Vancomycin, levofloxacin, metronidazole, ceftriaxone | 74 | M | R | K | 0 | 1 | 0 | 6.3 | 53 | 50.7 | 79689 | 94 | 1 | Granulation tissue with acute inflammation |
| 69 | 0/3 | 0 | 23076439 | Negative | | 1 | 1 | Minocycline | 60 | M | R | K | 1 | 1 | 0 | 8.1 | 13 | 36.5 | 18509 | 87 | nd | |

| | | | | | | | | | | | | | | | | | | | | | | |
|---|---|---|---|---|---|---|---|---|---|---|---|---|---|---|---|---|---|---|---|---|---|---|
| 70 | 0/5 | 0 | 29693924 | *Staphylococcus aureus* | 67 | 1 | 2 | Cefdinir, rifampin | 66 | F | R | K | 0 | 1 | 0 | 4.5 | 23 | 4 | nd | nd | 0 | Negative for acute inflammation |
| 71 | Staphylococcus capitis 3/5, S. epidermidis 1/5 | S. epidermidis | 26726828 | *Staphylococcus capitis. Staphylococcus epidermidis* | 139 | 1 | 1 | Doxycycline, tetracycline | 62 | M | L | H | 1 | 1 | 1 | 8.2 | 30 | 22.2 | 3053 | 79 | 0 | Synovium with granulation tissues |
| 72 | S. aureus 3/3 | Staphylococcus aureus | 30488780 | *Staphylococcus aureus* | 38 | 1 | 3 | Cephalexin, rifampin | 73 | M | L | K | 1 | 1 | 0 | 6.3 | 73 | 110.4 | 155636 | 97 | 1 | Granulation tissue with acute inflammation and associated fibrotic tissue |
| 73 | Candida parapsilosis 3/3, C. acnes 2/3 | Bacillus cereus | 28731233 | *Candida parapsilosis， C. acnes* | 114 | 1 | 2 | Cephalexin, fluconazole | 64 | M | R | K | 1 | 1 | 0 | 6.6 | 34 | 9.8 | nd | nd | 1 | Focal acute inflammation |
| 74 | Streptococcus mitis 5/5 | 0 | 31682445 | *Streptococcus gordonii* | 25 | 1 | 1 | Unknown | 77 | F | L | K | 1 | 2 | 1 | 9.2 | 19 | 13 | 2316 | 58 | 1 | Soft tissue with chronic inflammation, no acute inflammation seen |
| 75 | 0/4 | 0 | 26375749 | Negative | | 1 | 3 | Cefadroxil, ampicillin/sulbactam, piperacillin/tazobactam | 55 | M | L | K | 1 | 2 | 0 | 9.1 | 19 | 9.8 | 5675 | 51 | 1 | Soft tissue with chronic inflammation |
| 76 | Streptococcus agalactiae 4/4 | Streptococcus agalactiae | 37499849 | *Streptococcus agalactiae* | 23 | 1 | 1 | Levofloxacin | 63 | F | R | H | 0 | 2 | 0 | 7.9 | 60 | 11.7 | 128439 | 75 | 1 | Positive for acute inflammation |
