# Supplementary material for: Will previous antimicrobial therapy reduce the positivity rate of metagenomic next-generation sequencing in periprosthetic joint infections? A clinical study
Source: Front Cell Infect Microbiol. 2024 Jan 11;13:1295962. doi: 10.3389/fcimb.2023.1295962 (PMC10808557; doi:10.3389/fcimb.2023.1295962)
Supplement: Supplementary file 3 [file DataSheet_3.pdf]

Non- and microbiologic results of cases without previous antimicrobial therapy

**Sample Number**	**Intraoperative Deep-tissue Culture Results** (# positive/total # collected)	**Intraoperative Synovial Fluid Culture Results** (0 = no growth)	**mNGS Results**	**Number of Reads**	**Preoperative Antibiotics within 4 weeks of surgery** (0=no, 1=yes)	**Age** (years)	**Sex**	**Side** (right or left)	**Joint** (K=knee H=hip)	**Acute Infection** (0=neg, 1=pos)	**Intraoperative Purulence** (0=no, 1=yes, 2=surgeon intraoperative impression of infection)	**Sinus Tract** (0=no, 1=yes, nk=not known)	**WBC** (10^3 cells/mm^3, nd=not done)	**Serum ESR** (mm/h, nd=not done, Ref range: 0-20)	**Serum CRP** (mg/L, nd=not done, Ref range: <=8.0)	**Total nucleated cell count** (cells/mm^3 nd=not done)	**% Neutrophils** (nd=not done)	**Histopathology** (0=neg, 1=pos, nd=not done)	**Histopathology description**
77	0/3	0	*Cutibacterium acnes*	181	0	56	M	L	K	0	1	0	9.9	35	45.7	29520	86	1	Synovial hyperplasia with chronic inflammation
78	C. acnes 2/4	0	*Parvimonas micra*	93	0	79	M	L	H	0	1	1	8.1	64	42	5770	79	0	Fibroconnective tissue with granulation tissue
79	0/4	0	*Cutibacterium acnes*	33	0	74	F	R	H	0	1	1	8.4	31	17.1	33864	93	1	Synovial tissue and significant acute inflammation
80	0/4	0	*Anaerococcus prevotii*	111	0	72	M	L	K	1	1	0	6.2	28	13.8	3495	61	1	chronic and minimal acute inflammation
81	Coagulase-negative Staphylococcus 2/4	Coagulase-negative Staphylococcus	*Staphylococcus epidermidis*	78	0	61	M	L	H	0	1	0	10.5	46	8.3	47189	98	1	Synovial tissue and significant acute inflammation
82	S. aureus 3/3	S. aureus	*Staphylococcus aureus*	301	0	34	M	R	H	0	1	0	14.7	114	235	nd	nd	0	AE, negative for acute inflammation

83	S. aureus 5/5	S. aureus	*Staphylococcus aureus*	23	0	93	F	R	H	0	1	0	30.1	84	335.9	432684	94	1	AE with acute inflammation
84	S. aureus 5/6	S. aureus	*Staphylococcus aureus*	812	0	75	M	L	K	0	1	0	7.1	46	23.2	32472	96	1	AE with acute inflammation
85	Streptococcus viridans 4/4	Streptococcus mitis	*Streptococcus oralis*	22	0	31	F	L	K	0	1	0	5.6	87	66.1	34275	81	nd	
86	S. aureus 3/3	Staphylococcus aureus	*Staphylococcus aureus*	34	0	51	M	R	H	0	1	0	8.5	94	110	45200	98	nd	
87	0/3	0	*Staphylococcus lentus*	476	0	78	M	R	K	0	0	0	6.6	25	5.5	12	19	1	Marked chronic proliferative synovitis with non-specific increased
88	Enterococcus faecalis 4/5, Coagulase-negative Staphylococcus 1/5	Enterococcus faecalis	*Enterococcus faecalis*	283	0	78	M	L	K	0	1	0	9.3	11	11.4	89	1	1	Mild chronic inflammation and fibrosis
89	0/5	0	*Corynebacterium jeikeium*	596	0	63	F	R	K	0	1	0	6.6	9	6.4	4319	77	1	Synovial hyperplasia with chronic inflammation

90	0/3	0	*Negative*		0	67	F	L	K	1	2	0	7.7	74	43.6	7975	55	1	Synovial tissue and acute inflammation
91	Staphylococcus capitis 2/4	0	*Staphylococcus capitis*, *Staphylococcus warneri*	467	0	74	M	L	K	0	1	0	6.5	60	58.2	27055	93	1	Synovial tissue and acute inflammation
92	Corynebacterium amycolatum 3/5	Gram-positive bacilli resembling Corynebacterium	*Corynebacterium amycolatum*	53	0	70	M	L	H	0	2	0	9	28	37.7	27060	92	nd	
93	0/3	0	*Staphylococcus lugdunensis*	1350	0	55	F	R	K	0	1	0	5	22	12.7	12587	92	1	Synovial tissue and acute inflammation
94	0/4	0	*Staphylococcus aureus*	589	0	90	F	L	K	1	0	0	12.5	29	22	8565	74	1	Synovial hyperplasia with chronic inflammation
95	0/4	0	*Negative*		0	60	M	R	K	0	2	0	5.6	1	4.4	3850	66	1	Synovial hyperplasia with chronic inflammation
96	0/4	0	*Granulicatella adiacens*	1643	0	76	F	R	K	0	1	0	3.6	75	53.3	60543	94	1	Synovial tissue and acute inflammation

97	0/3	0	*Negative*		0	31	F	L	K	0	0	0	6.8	8	9.4	4762	55	1	Synovial hyperplasia with chronic inflammation
98	S. epidermidis 4/4, E. faecalis 2/4	S. epi	*Enterococcus faecalis, Staphylococcus epidermidis*	4075	0	71	F	L	K	1	2	0	10.2	28	9	nd	nd	0	Hypertrophic synovium with fibrosis. Negative for acute inflammati
99	S. epidermidis 2/4	Staphylococcus epidermidis	*Staphylococcus epidermidis*	67	0	50	F	R	K	1	1	0	7.1	18	15.4	51250	91	1	Focal mild acute inflammation is present
100	S. aureus 3/3	S. aureus	*Staphylococcus aureus*	2696	0	70	M	L	K	1	1	0	6	44	135.5	114369	97	1	Granulation tissue with acute inflammation
101	S. epidermidis 4/4	S. epi	*Staphylococcus epidermidis*	519	0	64	M	R	K	0	0	0	8.7	58	38.4	30987	97	nd	
102	S. aureus 6/6	S. aureus	*Staphylococcus aureus*	96	0	65	F	R	H	1	1	0	13	71	196.8	397536	100	1	Dense fibrovascular tissue admixed with granulation tissue
103	S. epidermidis 3/3	S. epidermidis	*Staphylococcus epidermidis*	931	0	65	M	L	K	1	1	0	7.1	35	6.2	21405	93	1	AE, positive for acute inflammation

104	S. epidermidis 2/4	0	*Staphylococcus epidermidis*	315	0	77	M	R	K	0	1	0	6	35	39.4	46893	96	1	Acute inflammation
105	S. epidermidis 5/5, Finegoldia magna 4/5	0	*Finegolida magna. Staphylococcus epidermidis*	577	0	63	F	R	H	1	1	1	7.3	57	107.2	nd	nd	1	AE, positive for acute inflammation
106	0/3	Streptococcus salivarius	*Streptococcus salivarius*	85	0	66	M	L	K	0	1	0	7.9	50	7.4	11869	91	1	AE, positive for acute inflammation
107	0/6	0	*Mycobacterium tuberculosis*	326	0	84	F	L	H	1	0	0	7	42	10.6	50601	97	1	Areas of necrosis and associated mild acute inflammation
108	0/3	0	*C. acnes*	56	0	69	M	L	K	0	0	0	5.5	6	12.8	2160	30	1	Marked mixed chronic inflammation is present
109	C. acnes 3/3	0	*C. acnes*	845	0	71	M	R	H	0	0	0	6.3	7	6.1	312	96	0	Dense fibrous tissue, negative for acute inflammation
110	0/4	0	*Pseudomonas aeruginosa*	195	0	75	F	L	H	0	0	0	10.7	71	33.8	1712	79	1	Synovial hyperplasia with chronic inflammation

111	S. epidermidis 3/3	S. epidermidis	*Staphyolcoccus epidermidis*	60	0	52	F	L	K	1	1	0	7.6	33	32	70665	97	1	Synovium with foci of acute inflammation and dense fibroconne
112	0/6	0	*Negative*		0	72	M	R	H	0	0	0	8	40	13	772	88	1	Synovial hyperplasia with chronic inflammation
113	0/5	0	*Negative*		0	45	M	R	H	0	1	0	7.7	13	7.3	nd	nd	1	Synovial hyperplasia with chronic inflammation
114	Pseudomonas aeruginosa 1/7	0	*Negative*		0	80	F	L	H	1	1	0	4.9	31	14.7	1768	11	0	Fibrous tissue with rare neutrophils present
115	S. epidermidis 4/4	0	*Staphylococcus epidermidis*	204	0	62	M	R	H	1	1	0	7.5	37	56.8	3036	85	1	Synovial hyperplasia with chronic inflammation
116	S. agalactiae 5/5	S. agalactiae	*Streptococcus agalactiae*	54	0	42	M	R	H	1	1	0	12.6	19	180.7	21112	94	1	Positive for acute inflammation
117	0/5	0	*Negative*		0	64	M	R	K	0	2	0	6.3	14	11.4	287	28	1	Synovial hyperplasia with chronic inflammation

118	0/3	0	*Pseudomonas oleovorans*	56	0	80	F	R	K	0	2	0	7.3	85	49.7	nd	nd	1	Positive for acute inflammation
119	P. avidum 1/4, Bacillus cereus 1/4	0	*Propionibacterium avidum*	18	0	59	M	R	H	0	0	0	6.4	9	11.5	20064	88	1	Positive for acute inflammation
120	E. faecalis 1/4	0	*Enterococcus faecalis Staphylococcus haemolyticus*	145	0	71	F	L	H	0	1	0	6.3	69	24.5	2080	49	1	Synovial hyperplasia with chronic inflammation
121	Candida albicans 1/4	Candida albicans	*Candida albicans*	106	0	72	M	L	H	0	2	0	9.3	19	87.7	82820	79	1	Fibroartciular tissue with patchy acute and chronic inflammation
122	S. epidermidis 3/3	S. epidermidis	*S. epidermidis, A johnsonii, C. acnes*	346	0	68	F	R	K	0	2	0	7.4	115	14.8	16223	85	1	Positive for acute inflammation
123	0/5	0	*Mycoplasma salivarium*	532	0	53	M	R	K	1	1	1	12.6	28	91.1	28756	93	1	Positive for acute inflammation
124	0/5	0	*Corynebacterium pyruviciproducens*	743	0	67	F	R	K	0	1	0	7.1	32	49.7	36562	70	nd	

125	0/3	0	*Streptococcus agalactiae*	40	0	47	F	L	K	0	2	0	4.3	51	15.3	35055	94	nd	
126	Streptococcus mitis group 3/3, Anaerococcus hydrogenalis 1/3	Streptococcus mitis gp	*Streptococcus sanguinis Anaerococcus hydrogenalis*	266	0	55	F	L	K	1	1	1	8.5	32	67.7	82	8	0	Granulation tissue with occasional neutrophils
127	0/4	S. epidermidis, S. lugdunensis	*S. epidermidis, S. lugdunensis*	38	0	58	F	L	H	0	1	0	3.1	2	3.4	nd	nd	1	Synovial hyperplasia with chronic inflammation
128	0/5	0	*Finegoldia magna*	61	0	58	F	L	H	0	2	0	5.4	5	3.1	5761	85	0	Fibrosis. Negative for acute inflammation
129	S. epidermidis 2/3	S. epidermidis	*Staphylococcus epidermidis*	692	0	66	M	L	K	1	1	0	6.1	9	10.6	nd	nd	1	Arthroplasty effect with acute inflammation
130	Mycobacterium bovis 3/3	0	*Mycobacterium tuberculosis* complex	189	0	75	M	R	H	0	0	0	8.3	62	31.4	nd	nd	1	Arthroplasty effect with acute inflammation
131	G. adiacens 1/3	0	*Granulicatella adiacens*	479	0	47	M	R	H	0	2	0	6.7	27	17	20210	98	nd	
